# Phenotypic and Functional Alterations in Circulating Memory CD8 T Cells with Time after Primary Infection

**DOI:** 10.1371/journal.ppat.1005219

**Published:** 2015-10-20

**Authors:** Matthew D. Martin, Marie T. Kim, Qiang Shan, Ramakrishna Sompallae, Hai-Hui Xue, John T. Harty, Vladimir P. Badovinac

**Affiliations:** 1 Interdisciplinary Graduate Program in Immunology, University of Iowa, Iowa City, Iowa, United States of America; 2 Department of Pathology, University of Iowa, Iowa City, Iowa, United States of America; 3 Department of Microbiology, University of Iowa, Iowa City, Iowa, United States of America; 4 Iowa Institute of Human Genetics Bioinformatics Division, University of Iowa, Iowa City, Iowa, United States of America; St. Jude Children's Research Hospital, UNITED STATES

## Abstract

Memory CD8 T cells confer increased protection to immune hosts upon secondary viral, bacterial, and parasitic infections. The level of protection provided depends on the numbers, quality (functional ability), and location of memory CD8 T cells present at the time of infection. While primary memory CD8 T cells can be maintained for the life of the host, the full extent of phenotypic and functional changes that occur over time after initial antigen encounter remains poorly characterized. Here we show that critical properties of circulating primary memory CD8 T cells, including location, phenotype, cytokine production, maintenance, secondary proliferation, secondary memory generation potential, and mitochondrial function change with time after infection. Interestingly, phenotypic and functional alterations in the memory population are not due solely to shifts in the ratio of effector (CD62Llo) and central memory (CD62Lhi) cells, but also occur within defined CD62Lhi memory CD8 T cell subsets. CD62Lhi memory cells retain the ability to efficiently produce cytokines with time after infection. However, while it is was not formally tested whether changes in CD62Lhi memory CD8 T cells over time occur in a cell intrinsic manner or are due to selective death and/or survival, the gene expression profiles of CD62Lhi memory CD8 T cells change, phenotypic heterogeneity decreases, and mitochondrial function and proliferative capacity in either a lymphopenic environment or in response to antigen re-encounter increase with time. Importantly, and in accordance with their enhanced proliferative and metabolic capabilities, protection provided against chronic LCMV clone-13 infection increases over time for both circulating memory CD8 T cell populations and for CD62Lhi memory cells. Taken together, the data in this study reveal that memory CD8 T cells continue to change with time after infection and suggest that the outcome of vaccination strategies designed to elicit protective memory CD8 T cells using single or prime-boost immunizations depends upon the timing between antigen encounters.

## Author Summary

Following infection or vaccination, memory CD8 T cells persist at higher numbers and have enhanced functional abilities compared to naïve cells, providing immune hosts with increased protection from viral, bacterial, or parasitic infection. Protection provided by memory CD8 T cells depends on the numbers, quality (functional abilities), and location of cells present at the time of re-infection. While memory CD8 T cells can be maintained for great lengths of time, how time influences qualitative properties of these cells remains largely unknown. We show that the phenotype and functions of circulating memory CD8 T cells, including cytokine production, proliferation, and mitochondrial function following re-infection improves with time after infection. We also show that changes in function are not due solely to changes in subset composition of the memory pool. Importantly, due to enhanced proliferative and metabolic abilities, memory CD8 T cells analyzed late after infection were more protective against a chronic viral infection. Our study shows that the properties of memory CD8 T cells continue to change with time, and that the protective outcome of vaccination may depend on the timing of re-infection relative to the initial immunization.

## Introduction

Memory CD8 T cells provide immune hosts with enhanced protection from pathogenic infection due to an increased precursor frequency of antigen (Ag)-specific cells, widespread localization to both lymphoid and non-lymphoid tissues, and ability to rapidly execute effector functions such as cytokine production and cytolysis compared to naïve CD8 T cells [1–3]. Protection provided by memory CD8 T cells is dependent upon the number, quality (functional abilities), and location of memory CD8 T cells available at the time of infection. Importantly, the quality and location of memory CD8 T cells best suited to combat diverse infections is dependent upon the tropism of the invading pathogen. Memory CD8 T cells consist of a heterogeneous population of cells [4] that were initially categorized into central memory (T_cm_) and effector memory (T_em_) subsets based on CCR7 and CD62L expression, and that differ in anatomical location and functionality [5,6]. Recently, an additional subset of memory CD8 T cells has been described that reside in non-lymphoid tissues and that have been called tissue-resident memory (T_rm_) cells [7]. While the relative protection provided by circulating T_cm_ and T_em_ cells differs depending on the nature of infection [6,8–10], both are better suited to provide protection against systemic infection than T_rm_ cells that provide enhanced protection against infection that occurs within peripheral tissues [11–15]. Several studies have suggested that T_rm_ cells may be long-lived in the skin following VacV or HSV infection and in mucosal surfaces following intramuscular immunization with adenovirus vectors [12,15,16]. However, other studies examining T_rm_ generated following influenza have suggested that T_rm_ cell numbers wane following infection [17]. Therefore, longevity of T_rm_ cells likely depends on the infection/vaccination model and the tissue of memory residence. However, circulating memory CD8 T cells persist for great lengths of time following immunization or systemic viral infection. For example, lymphocytic choriomeningitis virus (LCMV)-specific memory CD8 T cells are maintained at stable numbers in the spleen for the life of the laboratory mouse [18], and detectable numbers of memory CD8 T cells can be found in human PBL 20–75 years after natural exposure to, or vaccination against yellow fever virus, measles virus, and smallpox [19–23]. However, several studies have indicated that some properties of circulating memory CD8 T cells change with time after infection. For example, expression of CD62L and CD27 (markers of central memory cells) increases, indicating that the subset composition of the memory population changes with time after infection. In addition, functions such as cytokine production, proliferation, and memory generation following Ag re-encounter, increase with time [24–28]. The full extent of phenotypic and functional alterations that occur within the memory CD8 T cell population with time after infection, however, remains poorly characterized. It is unclear if alterations are due solely to differences in subset composition of memory CD8 T cell populations, or to changes within defined memory subsets. These are important questions to address, as the level of protection provided against systemic infections may change with time following initial infection and/or vaccination.

While most vaccines are intended to elicit protection against seasonal illnesses or pathogens that will be encountered in the relatively near future, infection may not occur for long periods of time following the original vaccination. Therefore, changes in memory CD8 T cell function between vaccination and the time of infection may impact the protection provided by memory CD8 T cells. Furthermore, evidence has suggested that the number of memory CD8 T cells required to provide protection against some pathogens may be quite high [29–31]. Currently, the best method for eliciting large numbers of memory CD8 T cells involves prime-boost strategies in which a series of vaccinations are administered allowing for a period of time between boosts [32]. Recently, it was reported that all human subjects receiving five injections of cryopreserved radiation attenuated *Plasmodium falciparum* sporozoites were protected upon controlled infection with malaria, while not all subjects receiving four injections were protected [31]. A number of potential reasons for the increased protection provided by the five-dose regimen were proposed including a longer interval of time between administration of the fourth and fifth boost. Although not tested in their work, the authors argued that increasing the interval of time between boosts could lead to the establishment of greater numbers of memory CD8 T cells and increased protection compared to immunization strategies using a shorter time interval between boosts. Thus, functional changes in the properties of memory CD8 T cells occurring in the time between boosts may directly impact the protection achieved through prime-boost vaccination strategies. For these reasons, an understanding of how memory CD8 T cell quality changes with time after infection and/or vaccination is needed.

In this study we examined how the properties of circulating memory CD8 T cells change with time following an acute systemic infection with LCMV. We demonstrate that memory CD8 T cell quality changes with time after Ag-encounter in a manner not solely due to shifts in memory subset composition. Importantly, our data suggests that alterations in memory CD8 T cell function that occur with time after Ag-encounter could impact their ability to provide protection against diverse pathogens, and that the generation of memory CD8 T cells through prime boost protocols may depend on the timing between boosts.

## Results

### Changes occur in memory CD8 T cell location, phenotype, function, and maintenance with time after infection

Heterogeneous populations of Ag-specific CD8 T cells can be analyzed on the level of the population (every CD8 T cell in the host), the subset level (CD8 T cells expressing a marker or combination of phenotypic markers), or the level of single cells. T_cm_ and T_em_ subsets differ in anatomical location and functionality [5,6]. Thus, differences in function between memory populations could be due to alterations in subset composition that occur with time after primary antigen recognition. Additionally, individual cells within the population and within subsets can differ in phenotype and function from one another. However, because the level of protection is determined by the quality of all memory CD8 T cells present at the time of re-infection, we first examined how circulating memory CD8 T cells change with time after infection when analyzed on the population level. We adoptively transferred low numbers of naïve Thy1.1 or Thy1.1/1.2 transgenic (Tg) P14 CD8 T cells specific for the glycoprotein (GP)_33-41_ epitope derived from LCMV into Thy1.2 C57BL/6 recipients and infected recipients with LCMV 24 hours (h) later. We then analyzed memory P14 cells on the population level (i.e. all memory cells present in the examined organs) 30–45 days (*early*M) or 8+ months (*late*M) later.

**Fig 1:**
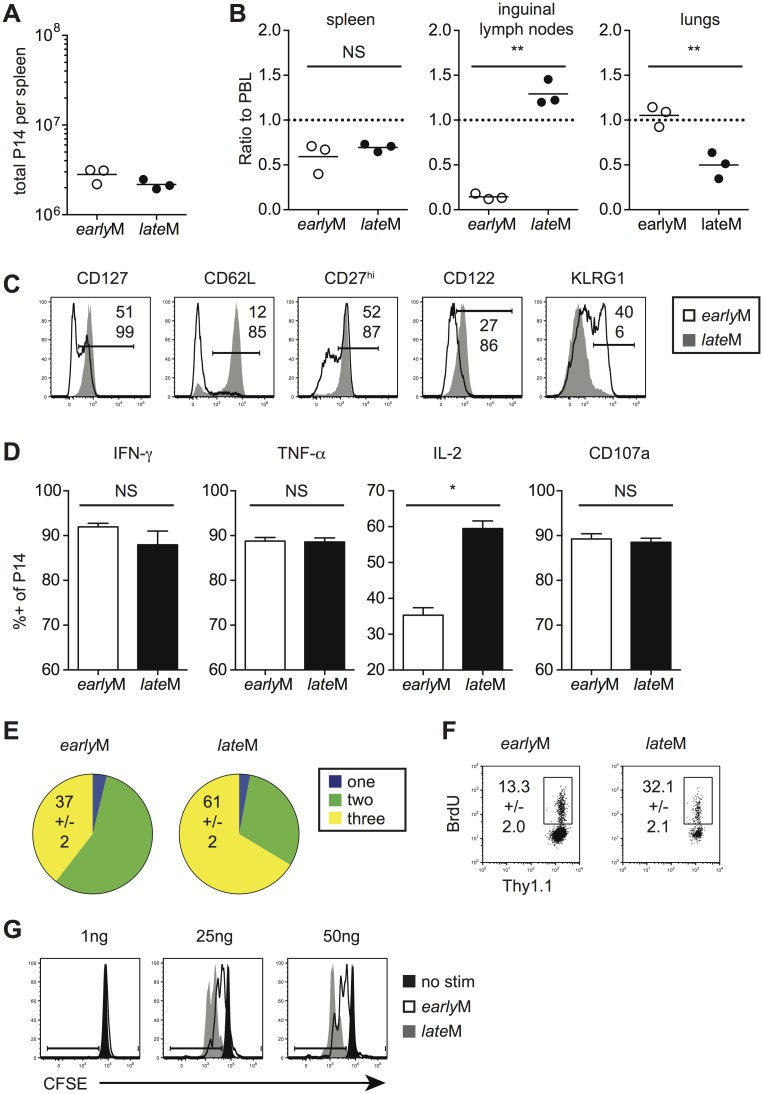
Localization, phenotype, and function of memory CD8 T cells changes with time after infection. Memory P14 cells were generated by adoptively transferring 5x10^3^ naïve P14 T cells into Thy disparate naïve recipients followed by i.p. injection of LCMV 24 hours later. Analysis was performed on *early*M (30–45 days p.i.) and *late*M (8+ months p.i.) P14 cells. (A) Numbers of *early*M and *late*M P14 cells found in the spleens of recipient mice. (B) Ratios of the percentage of *early*M or *late*M P14 cells in the indicated organs following perfusion of tissues out of total lymphocytes to the percentage of *early*M or *late*M P14 cells in PBL out of total lymphocytes. (C) Representative histograms showing CD127, CD62L, CD27, CD122, and KLRG1 expression on gated *early*M (open histograms) and *late*M (grey histograms) P14 cells isolated from spleens. (D) Percentage of gated *early*M or *late*M P14 cells producing IFN-γ, TNF-α, or IL-2 as measured by ICS, or undergoing degranulation as measured by surface CD107a expression following 5hr incubation with GP_33-41_ peptide. (E) Percentage of gated *early*M or *late*M P14 cells producing one (IFN-γ), two (IFN-γ and TNF-α), or three (IFN-γ, TNF-α, and IL-2) cytokines as measured by ICS following 5hr incubation with GP_33-41_ peptide. (F) Representative dot plots of BrdU staining for gated *early*M or *late*M P14 cells in PBL 8 days after BrdU injection. (G) Representative histograms of CFSE dilution on gated *early*M (open histograms) or *late*M (grey histograms) P14 cells after 3 day culture in the presence or absence (black histograms) of the indicated concentrations of IL-15. NS, not statistically significant; *, statistically significant (p<0.05); **, statistically significant (p<0.01) as determined by Student t-test. Representative data from one of three individual experiments with 3 mice per group per experiment. Error bars represent the standard error of the mean.

Although similar numbers were found in the spleen (Fig 1A), *early*M cells were found in greater proportions in the lungs while *late*M cells were found in greater proportions in the inguinal lymph nodes following perfusion of tissues (Fig 1B). Surface marker profiles also differed with time, with expression of CD127, CD62L, CD27, and CD122 increasing and expression of KLRG1 decreasing with time after infection (Fig 1C). Similar patterns of surface marker expression were also seen for endogenous *early*M and *late*M GP_33_ and GP_276 –_specific CD8 T cell responses (S1A Fig). To determine if cytokine production or degranulation changes with time, intracellular cytokine staining (ICS) was performed on *early*M and *late*M cells that were mixed and incubated with GP_33-41_ peptide for 5 h. The percentages of IFN-γ and TNF-α producing P14 cells and degranulation as measured by surface CD107a expression did not change with time. However, the percentage of memory CD8 T cells able to produce IL-2 increased with time (Fig 1D). Consistent with these data, the percentage of cells capable of polyfunctional cytokine production (IFN-γ, TNF-α, and IL-2) increased with time after infection (Fig 1E). Finally, to determine if maintenance of memory CD8 T cells changes with time, basal proliferation of *early*M and *late*M cells was examined with bromodeoxyuridine (BrdU) incorporation during an eight-day period. Interestingly, a higher percentage (p<0.01) of *late*M cells incorporated BrdU during the eight-day interval (Fig 1F), indicating that the rate of basal proliferation increases in memory CD8 T cells with time after infection. Basal proliferation is partially dependent upon IL-15 signaling [33–35], and increased expression of IL-15Rβ (CD122) in *late*M cells (Fig 1C) suggested that sensitivity to IL-15 could change with time. To test this, Thy disparate *early*M and *late*M cells were labeled with carboxyfluorescein diacetate succinamidyl ester (CFSE), mixed and incubated with increasing concentrations of IL-15, and dilution of CFSE was determined after 3 d. *late*M cells proliferated to a greater extent in response to exogenous IL-15 and were more sensitive to lower levels of IL-15 (Fig 1G), indicating that sensitivity to IL-15 increases over time. Expression of CD122 was also greater in *late*M compared to *early*M endogenous GP_33_ and GP_276_ tetramer positive cells (S1A Fig), suggesting that endogenous *late*M cells are also more responsive to IL-15 compared to *early*M cells. These data show that while memory CD8 T cells analyzed on the population level persist at stable numbers in the spleen after infection, time changes their location, surface marker expression, Ag-driven cytokine production, and ability to respond to homeostatic clues in the environment.

### Antigen-driven proliferation and memory generation potential increase in memory CD8 T cells over time

**Fig 2:**
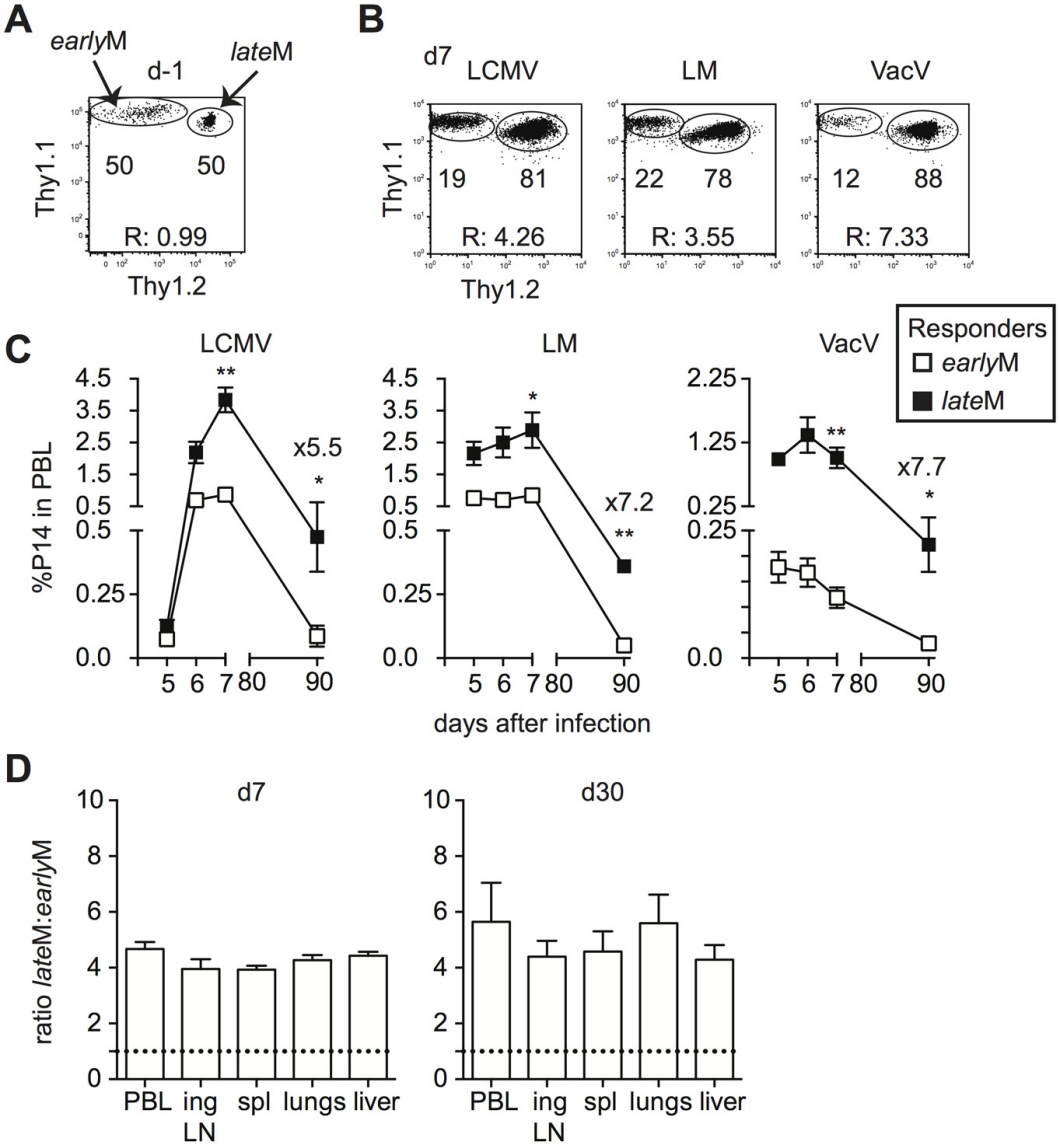
Proliferation and ‘memory generation potential’ in response to Ag-reencounter increases with time after infection. Thy disparate *early*M and *late*M P14 cells were mixed and injected into naïve recipients followed by i.p. injection of LCMV Armstrong (LCMV) or VacV, or i.v. injection of LM 24 hours later. (A) Dot plot showing the master mix of *early*M (Thy1.1/1.1) and *late*M (Thy1.1/1.2) P14 cells used for adoptive transfer. (B) Representative plots showing the response of progeny of *early*M (Thy1.1/1.1) and *late*M (Thy1.1/1.2) P14 cells in PBL d7 after the indicated infections. (C) Kinetic analysis of secondary responses generated from *early*M and *late*M P14 cells in PBL at the indicated days post LCMV, LM, or VacV infection. (D) Ratio of *late*M to *early*M cells generated from transferred P14 cells in the indicated organs after LCMV infection at the height of the effector response (d8- left) or at a memory time point (d30-right). Doted lines indicate the starting ratio of *late*M to *early*M P14 cells. R:, ratio of *late*M to *early*M P14 cells. *, statistically significant (p<0.05); **, statistically significant (p<0.01) as determined by Student t-test. Representative data from one of three individual experiments with 5 mice per group per experiment. Error bars represent the standard error of the mean.

Primary memory CD8 T cells robustly proliferate and generate secondary effector and memory populations after Ag re-encounter [27,36]. Proliferation by primary memory CD8 T cells was reported to increase with time after initial Ag exposure following secondary infection with Sendai virus or *Listeria monocytogenes* [26,27]. To verify that time-dependent changes in Ag-driven proliferation and secondary memory generation of primary memory CD8 T cells analyzed on the population level are not dependent upon the type of infection or Ag-specificity, we set up adoptive co-transfer experiments. Thy disparate *early*M and *late*M P14 cells were mixed (Fig 2A) and 2x10^4^ of each were transferred into naïve C57BL/6 recipients followed by infection with various pathogens expressing GP_33_. This experimental setup ensured that secondary responses generated from *early*M and *late*M cells were subject to the same *in vivo* environmental conditions throughout the response. Secondary responses generated from *early*M and *late*M P14 cells after LCMV Armstrong (LCMV), LM, and Vaccinia Virus (VacV) infection were tracked longitudinally in peripheral blood (PBL), and a significantly greater percentage of secondary effector cells was found for responses generated from *late*M compared to *early*M P14 cells after each infection (Fig 2B and 2C). Additionally, when progeny were examined at a secondary memory time point, we found a 5–7 fold increase in the percentage of secondary memory cells generated from *late*M compared to *early*M P14 cells after each infection (Fig 2C). Furthermore, a greater percentage of secondary effector and memory cells was found for responses generated from *late*M compared to *early*M P14 cells in multiple organs following LCMV infection (Fig 2D). These results indicate that reduced progeny generated from *early*M cells was not due to restriction of responses by primarily CD62L- *early*M cells to non-lymphoid tissues. Taken together, these data indicate that Ag-driven proliferation and memory generation potential of primary memory CD8 T cells analyzed on the population level increases with time after infection irrespective of antigen specificity or type of infection.

### Phenotypic heterogeneity of CD62Lhi memory CD8 T cells decreases with time

Historically, circulating memory CD8 T cells have been characterized as CD62Llo T_em_ or CD62Lhi T_cm_ cells based on expression of CD62L and localization in peripheral tissues and lymphoid organs [5,6]. Functional differences between T_em_ and T_cm_ subsets also have been demonstrated, with T_cm_ having a greater capacity to produce IL-2, increased proliferative potential, and the ability to provide increased protection following some, but not all infections [6,8]. Therefore, the functional changes observed in the memory CD8 T cell population with time could be due solely to changes in subset composition. Alternatively, the properties of defined subsets of memory CD8 T cells could change with time.

To test the hypothesis that changes in memory CD8 T cell properties with time after infection are not due solely to shifts in subset composition, we began to examine the properties of *early*M and *late*M CD62Lhi cells. We chose to focus on CD62Lhi memory subsets for two reasons. First, with time after acute infection memory CD8 T cells convert to a population that is primarily CD62Lhi [24,25,27,28] (S2A Fig). Second, with time the CD62Llo population becomes enriched for T death intermediate memory cells (T_DIM_s) that arise from homeostatic division, are non-functional, and are destined to die [37]. To further document this, we examined IFN-γ production by CD62Lhi and CD62Llo *early*M and *late*M P14 cells following stimulation with cognate Ag. The capacity of CD62Lhi *early*M and *late*M cells to produce IFN-γ was similar (S2B Fig). In contrast, a higher percentage of CD62Llo *late*M compared to *early*M cells were unable to produce IFN-γ (S2B Fig), consistent with an enrichment of T_DIM_s. At present, there is no reliable phenotypic marker that can be used to distinguish T_DIM_s from normally functioning memory CD8 T cells within the CD62Llo population. Thus, by focusing our analysis on CD62Lhi memory CD8 T cells, we were able to examine how a well-characterized subset of memory CD8 T cells changes with time after infection while avoiding complications in analyzing CD62Llo populations.

**Fig 3:**
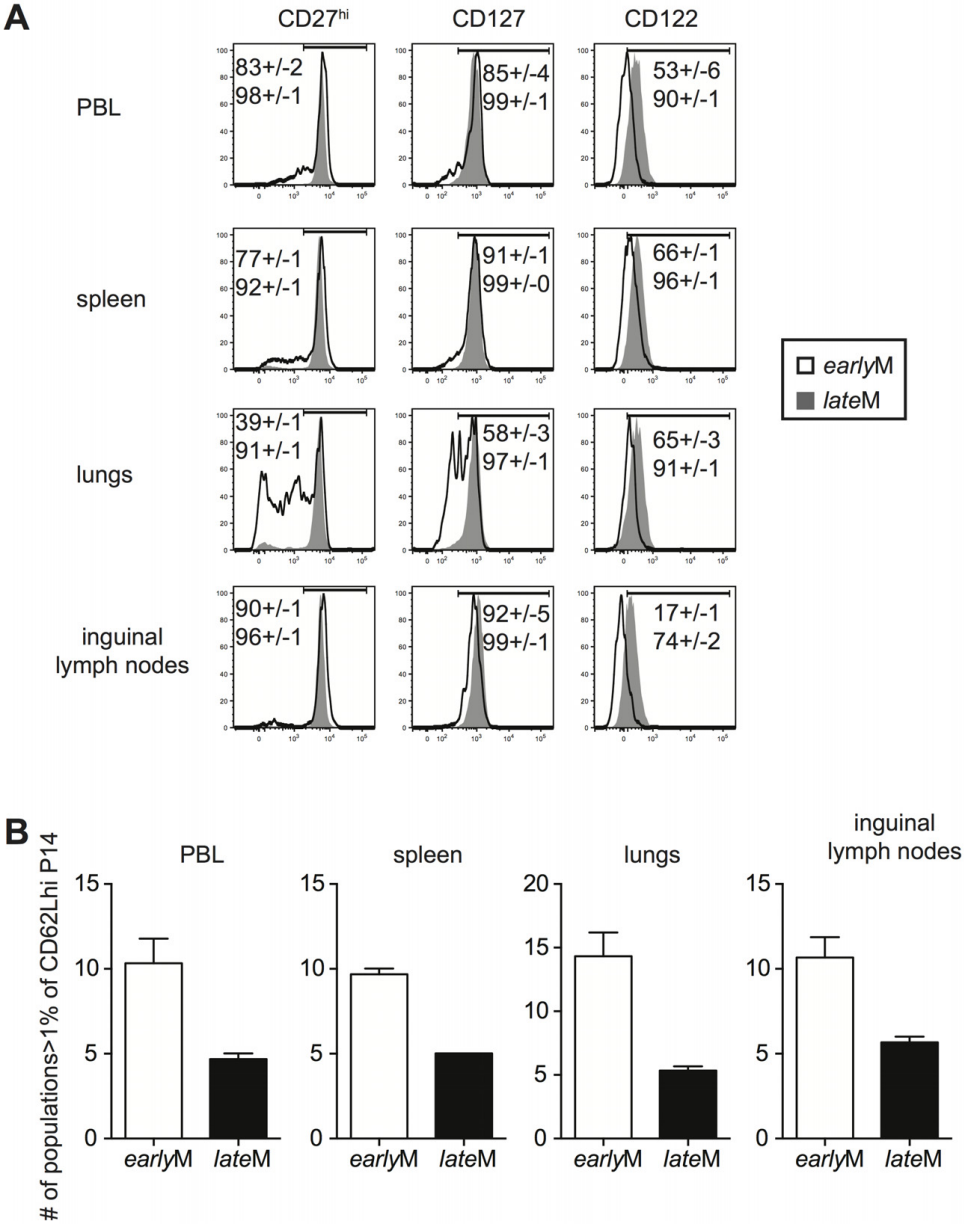
Phenotypic heterogeneity of memory CD8 T cells decreases with time after infection. *early*M (30–45 days p.i.) and *late*M (8+ months p.i.) P14 cells from the indicated organs were co-stained for CD62L, CD27, CD122, CD127, KLRG1, and CD11b. (A) Representative histograms of CD27, CD127, and CD122 expression on gated CD62Lhi *early*M (open histogram) and *late*M (grey histogram) P14 cells in the indicated organs. Numbers inside histograms indicate the percentage of CD62Lhi *early*M (top) or *late*M (bottom) cells staining positive for the indicated marker. (B) Number of subpopulations (out of 32 possible) comprising greater than 1% of the total CD62Lhi P14 population for *early*M and *late*M P14 cells in the indicated organs. Representative data from one of three individual experiments with 3 mice per group per experiment. Error bars represent the standard error of the mean.

Surface marker expression has been used to characterize memory CD8 T cells with different functional abilities. We rationalized that if CD62Lhi *early*M and *late*M cells displayed changes in phenotype, they also were likely to display other differences as well. Therefore, we began to explore to what extent the phenotype of CD62Lhi memory populations was influenced by time by examining the expression of CD27, CD127, and CD122, markers highly expressed by T_cm_ cells, on CD62Lhi *early*M and *late*M P14 cells (Fig 3A). The percentage of CD62Lhi cells expressing CD27, CD127, and CD122 was greater in *late*M compared to *early*M P14 cells in the peripheral blood (PBL) and spleen indicating that the phenotype of CD62Lhi memory CD8 T cells continues to change with time. This pattern was also seen for expression of CD122 and KLRG1 on gated CD62Lhi endogenous GP_33_ and GP_276_ *early*M and *late*M cells (S1B Fig). The percentage of cells expressing CD27, CD127, and CD122 among CD62Lhi *early*M P14 cells also differed between organs. Percentages of cells expressing CD27 and CD127 in the lungs were lower compared to the PBL, spleen, and inguinal lymph nodes, while percentages of cells expressing CD122 were lower in the inguinal lymph nodes compared to the PBL, spleen, and lungs. In contrast, percentages of CD62Lhi *late*M cells expressing CD27, CD127, and CD122 were uniformly high and were similar regardless of anatomical location except for a reduction in the percentage of cells expressing CD122 in the inguinal lymph nodes. This suggested that phenotypic heterogeneity within the CD62Lhi memory CD8 T cell subset decreases with time after infection.

To further test this, we co-stained for expression of CD27, CD127, CD122, CD11b, and KLRG1 on CD62Lhi *early*M and *late*M P14 cells. This strategy allowed us to differentiate 32 different subpopulations of CD62Lhi memory P14 cells, and the percentage of each subpopulation of total CD62Lhi *early*M and *late*M P14 cells is shown in S3A Fig. To examine heterogeneity within CD62Lhi *early*M and *late*M cells, the number of subpopulations (out of 32) comprising greater than 1% of the total CD62Lhi memory pool was counted. In each of the organs examined, *early*M contained 2–3 fold greater numbers of subpopulations than *late*M cells (Fig 3B). Similar patterns were seen for endogenous GP_33_ and GP_276_ tetramer positive memory cells (S1C and S1D Fig), and for CD62Lhi *early*M and *late*M P14 cells co-stained with CD27, CD127, CD43, CxCr3, and CCR5 (S3B Fig). Taken together, these data indicate that the phenotype of CD62Lhi memory CD8 T cells continues to change while heterogeneity of memory CD8 T cells decreases with time after infection.

### Gene expression patterns among CD62Lhi memory CD8 T cells change with time

**Fig 4:**
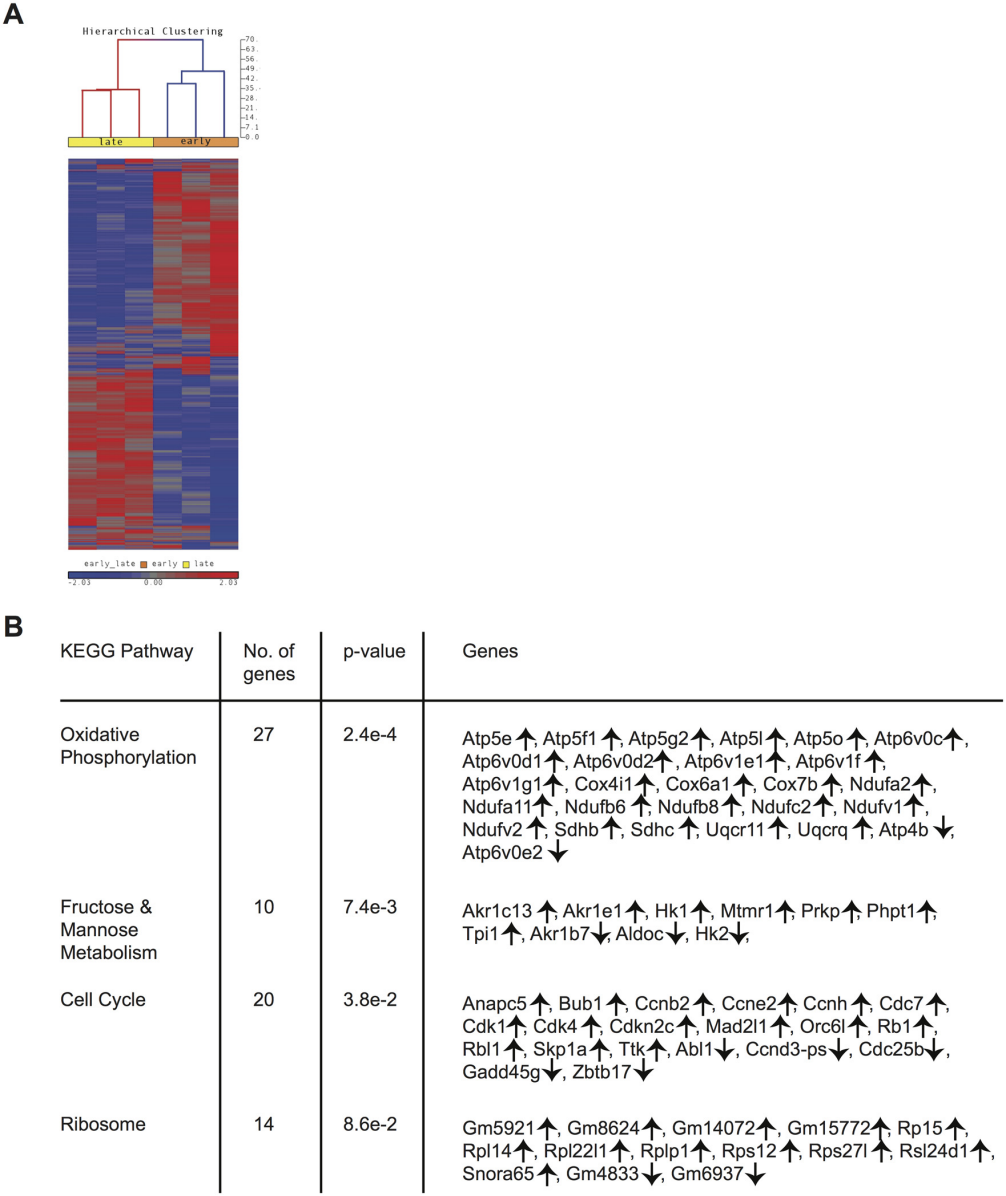
Gene expression patterns among CD62Lhi memory CD8 T cells change with time after infection. mRNA was isolated from sorted CD62Lhi *early*M (30–45 days p.i.) and *late*M (8+ months p.i.) P14 cells and used for microarray hybridization. (A) Heat map of genes with significantly different mRNA expression between *early*M and *late*M CD62Lhi P14 cells with a fold change >1.25. (B) Biological pathway analysis of genes with significant mRNA changes of fold >1.25 in CD62Lhi *early*M and *late*M P14 cells was generated using the KEGG pathway tool in DAVID bioinformatics resources. ↑, genes with increased expression in CD62Lhi *late*M compared to *early*M P14 cells. ↓, genes with decreased expression in CD62Lhi *late*M compared to *early*M P14 cells. Data were generated from six separate microarray hybridizations performed using mRNA extracted from sorted *early*M and *late*M P14 cells from three individual mice per group.

Differences in phenotype among CD62Lhi *early*M and *late*M P14 cells suggested that gene expression and functional changes could also occur with time. In order to determine the extent of changes in gene expression that occur with time within CD62Lhi memory subsets and to determine if genes regulating memory CD8 T cell functions were differently expressed, we examined the transcriptomes of CD62Lhi *early*M and *late*M P14 cells. We detected 3,494 genes that were differentially expressed (p<0.05) between CD62Lhi *early*M and *late*M P14 cells, and Fig 4A shows a heat map of genes with significantly different expression between CD62Lhi *early*M and *late*M P14 cells at fold differences >1.25. To determine if gene families with function in T cell biology are differentially expressed between CD62Lhi *early*M and *late*M P14 cells, we used DAVID bioinformatics resources [38] to assign biological functions and to group genes into function-related classes for genes with significantly different expression (p<0.05) and fold >1.5. This analysis revealed that mRNA expression for genes involved in many cellular processes are differentially expressed over time after infection in CD62Lhi memory cells (Table 1). Prominently among these changes, CD62Lhi *late*M cells showed increased expression of cytokine receptors including *Il2rb* and *IL15ra* (encoding IL-2Rβ and IL-15Rα respectively), and decreased expression of killer-cell lectin-like receptors including *Klrg1*. Furthermore, *late*M cells showed alterations in genes regulating cell cycle progression and ribosome biogenesis, suggesting that the proliferative potential of memory CD8 T cells may increase with time after infection not only at the population level (Fig 2), but also within defined subsets. Additionally, we used KEGG pathway analysis [39] to examine regulation of biological pathways in CD62Lhi *early*M and *late*M cells. This analysis revealed that multiple pathways are differentially regulated with time in CD62Lhi memory cells including cell cycle and ribosome pathways (Fig 4B). It is recognized that metabolic pathways are dynamically regulated during CD8 T cell responses, and both naïve and memory CD8 T cells catabolize fatty acid and utilize oxidative phosphorylation to meet their energy requirements [40–47]. The DAVID and KEGG analyses revealed that metabolism-related genes/pathways including metabolism of fatty acid, fructose and mannose metabolism, and oxidative phosphorylation were altered with time after infection in CD62Lhi memory CD8 T cells (Table 1 and Fig 4B). Taken together, these data further indicate that transcriptomic alterations occur within CD62Lhi memory CD8 T cells over time after infection.

**Table 1:** Functional annotation of genes significantly different between CD62Lhi *early*M and *late*M P14 cells at fold >1.5.

Gene	Alias	Fold	Gene	Alias	Fold	Gene	Alias	Fold	Gene	Alias	Fold
**Transcription**			**Cell Division, cell cycle**			**mRNA Processing**			**GTP binding**		
***AI987944***		**1.50**	***Bub1***		**1.56**	***Gemin6***		**1.66**	***Arl5a***		**1.68**
***Cep290***		**3.62**	***Casc5***		**1.76**	***Lsm5***		**2.52**	***Asna1***		**1.68**
***Cops5***		**1.87**	***Ccne2***		**1.85**	***Magohb***		**1.87**	***Gbp2***		**1.52**
***Dmrta1***	***Dmrt4***	**1.60**	***Cenpe***		**1.70**	***Nhp2l1***		**1.54**	***Gbp4***	***Mag-2***	**1.53**
***Eid3***		**1.70**	***Cetn2***	***Caltractiin***	**2.10**	***Pan2***		**1.63**	***Gm4841***		**1.51**
***Gm5595***		**1.64**	***Cks2***		**1.69**	***Ptbp2***		**1.61**	***Gnl3***		**1.54**
***Gm10778***		**2.66**	***Dscc1***		**1.93**	***Raly***		**1.51**	***Kti12***		**1.64**
***Gm13235***		**2.11**	***Erh***		**1.68**	***Sfs12ip1***		**1.51**	***Mx1***		**1.68**
***Ifl205***		**1.52**	***Gsg2***	***Haspin***	**1.61**	***Snrpd2***		**1.79**	***Rheb***		**1.58**
***Ikzf5***	***Pegasus***	**1.90**	***Kif11***		**2.25**	***Apobec2***		**-1.88**	***Sephs2***		**1.63**
***Kcnip3***		**2.12**	***Mad2l1***	***MAD2***	**1.71**				***Tgtp1***		**1.86**
***Med10***		**1.79**	***Nuf2***	***Cdca1***	**1.89**	**Ribosome**			***Uck2***		**2.07**
***Med28***		**1.59**	***Nusap1***		**1.90**	***Dkc1***		**1.61**	***Myo1e***		**-1.86**
***Pa2g4***	***Ebp1***	**1.63**	***Prc1***		**1.78**	***Gtpbp10***		**2.84**			
***Pcgf5***		**1.98**	***Rbl1***	***P107***	**1.68**	***LOC100044526***	***Gm17764***	**1.52**	**RNA binding**		
***Ppargc1a***		**1.86**	***Siah1a***		**1.51**	***Mrpl41***		**1.54**	***Cirbp***		**1.72**
***Prdm1***	***Blimp1***	**1.60**	***Smc2***		**1.80**	***Mrpl45***		**2.32**	***Ddx3x***		**2.10**
***Purb***		**1.94**	***Top2a***		**2.61**	***Mrpl46***		**1.52**	***Oasl2***		**1.74**
***Rybp***		**1.84**	***4632434I11Rik***		**2.19**	***Mrpl50***		**1.56**	***Paip2b***		**1.51**
***Smad1***	***Madh1***	**1.54**	***Vmn2r54***		**-1.58**	***Mrps18a***		**2.75**	***Raber2***		**1.59**
***Sabp2***		**1.72**				***Mrps18c***		**1.58**	***Tdrkh***		**1.57**
***Tada2a***		**1.53**	**Proteolysis**			***Mrps36***		**1.72**	***Ptms***		**-1.61**
***Taf12***		**1.55**	***Atg4a***		**1.71**	***Rps27l***		**1.60**	***Rbm47***		**-1.57**
***Tfb1m***		**1.57**	***Fbxo3***		**1.57**	***Snora65***		**1.60**			
***Thrap3***		**1.52**	***Fbxo30***		**1.66**	***Tsr2***		**1.51**	**Metal ion binding**		
***Zfp85-rs1***		**2.91**	***Qpct***		**1.96**				***Arsb***		**1.50**
***Zfp512***		**1.72**	***Smurf2***		**2.06**	**Golgi apparatus**			***BC005686***		**4.41**
***Zfp563***		**1.58**	***Trim32***	***Zfp117***	**1.97**	***Ap3m1***		**1.66**	***Cyp4f16***		**1.68**
***Zfp709***		**2.05**	***Ube2g2***		**2.12**	***Ap4b1***		**1.64**	***D10627***	***Zfp930***	**1.76**
***Zfp810***		**1.68**	***Ube2v2***		**2.19**	***Copz1***		**1.57**	***Efcab7***		**1.81**
***1300003B13Rik***	***Zfp946***	**1.74**	***Ufm1***		**1.73**	***Fam125b***		**1.77**	***Enpp2***		**1.68**
***2610044O15Rik***		**2.15**	***Capn11***		**-2.00**	***Fv1***		**2.24**	***Gca***		**1.66**
***2810021G02Rik***	***Zfp931***	**2.28**	***Csf1r***	***CD115***	**-1.58**	***Gosr2***	***SNARE***	**1.69**	***Harbi1***		**1.62**
***Bcl11a***		**-2.41**	***Gm5346***		**-1.76**	***Mgat4a***		**2.02**	***Hpcal1***		**1.60**
***Ciita***	***C2ta***	**-1.54**	***Lgmn***		**-1.86**	***Mudeng***		**1.88**	***Mid1***	***Trim18***	**2.75**
***Ebf1***		**-2.18**	***Napsa***	***pronapsin***	**-1.54**	***Rab1b***		**1.54**	***Mtrr***		**1.53**
***Hif1a***		**-1.84**				***Sar1a***		**1.62**	***Parp12***		**1.53**
***Spib***		**-1.80**	**Proteasome complex**			***Sec61b***		**1.56**	***Rnf160***	***Zfp294***	**1.83**
***Spic***		**-1.69**	***Psmb6***		**1.53**	***Stxbp5***		**1.58**	***S100a8***		**6.59**
***Tcf4***		**-1.75**	***Psmb7***		**1.59**	***Ust***		**1.51**	***Xdh***		**1.75**
***Zeb2***		**-1.73**	***Psmd8***		**1.61**	***Uxs1***		**1.51**	***Zbtb10***	***RINZF***	**3.25**
***Zfp69***	***Sip1***	**-2.30**	***Psmd9***	***P27***	**1.50**	***Vamp7***		**2.10**	***Aif1***		**-2.12**
						***Vps29***		**1.65**	***Baz2b***		**-1.78**
**Cytokines/Chemokines**			**Chaperone**			***Vps35***		**1.62**	***Capns2***		**-1.52**
***Ccl25***		**1.57**	***Atpaf2***		**1.83**	***1500035H01Rik***	***Ift46***	**1.53**	***Trf***		**-1.57**
***Fam3b***		**1.57**	***Bcs1l***		**1.55**	***Pgap1***		**-1.98**	***Zcchc3***		**-1.57**
***Il1b***		**1.72**	***Clpx***		**1.58**	***Slc15a2***		**-1.99**	***Ac3h12c***		**-1.67**
***Il7***		**2.04**	***Npm3***	***Nub1***	**1.71**	***St3gal1***		**-1.55**			
***Il15***		**2.36**	***Park7***		**1.82**	***St6gal1***		**-1.63**	**Miscellaneous**		
***Tnfsf8***	***CD30L***	**1.72**	***Pfdn2***		**1.82**				***Amn1***		**1.70**
***Tnfsf10***	***TRAIL***	**2.07**	***Vbp1***		**1.58**	**Extracellular region**			***Ankrd49***		**1.91**
***Xcl1***		**3.84**				***AI462493***		**1.61**	***AU017193***		**1.71**
***Ccl9***		**-1.74**	**Cell adhesion**			***Apol10b***		**1.81**	***AW549877***		**1.54**
			***Cdh1***	***Ecad***	**1.77**	***Bc028528***	***Ki67***	**-1.79**	***BC031781***	***Sde2***	**1.63**
**Cytokine receptors**			***Hspb11***		**1.68**	***Esm1***		**-1.61**	***Bex2***		**1.74**
***Il2rb***	***CD122***	**1.71**	***Itga1***	***CD49A***	**1.70**				***C030034I22Rik***		**2.73**
***Il6st***	***CD130***	**2.16**	***Lpp***		**1.70**	**Non-membrane organelle**			***Chic1***	***Brx***	**2.16**
***Il10ra***	***CD210***	**1.66**	***Mllt4***	***Afadin***	**1.79**	***Cenpc1***		**1.77**	***Csprs***	***HSR***	**1.56**
***Il15ra***	***CD215***	**1.68**	***Alcam***	***CD166***	**-1.99**	***Dynlt3***		**1.51**	***D230041D01Rik***		**1.66**
			***Cadm1***		**-1.66**	***Eml6***		**1.60**	***D330045A20Rik***		**1.96**
**Cytolytic effector molecules**			***Itga4***	***CD49D***	**-1.75**	***Gm11277***		**1.60**	***Dennd4a***		**2.09**
***Gzma***	***Ctla3***	**-6.43**	***Vcam1***	***CD106***	**-1.84**	***Mkl67***		**1.92**	***Dpy30***		**1.71**
						***Stmn1***		**2.58**	***Fam53b***		**1.70**
**Chemokine receptors**			**Chromatin modification**						***Fkbp2***		**1.57**
***Cx3cr1***		**-2.26**	***Hells***		**1.54**	**Membrane proteins**			***Fundc1***		**1.70**
***Cxcr5***		**-1.66**	***Sap18***		**1.53**	***Aqp9***		**2.43**	***G530011O06Rik***		**2.24**
						***Cldnd1***		**1.52**	***Gm129***		**1.77**
**Antigen Processing**			**Actin binding**			***Cnih4***		**1.56**	***Gm1419***	***Igkv4-68***	**1.52**
***Cd74***	***CLIP***	**-2.94**	***Arpc3***		**1.75**	***Dse***		**1.74**	***Gm4005***		**2.22**
***Fcer1g***	***FcRγ***	**-1.81**	***Cotl1***		**2.23**	***Enpp5***		**3.26**	***Gm5124***		**1.60**
***H2-Ab1***		**-3.03**	***Daam1***		**2.01**	***Fam165b***		**1.69**	***Gm527***		**1.84**
***H2-DMb2***		**-2.32**	***Tmsb15b1-Tmsb15b2***		**2.21**	***Mospd1***		**1.87**	***Gm7609***		**2.13**
***H2-Eb1***		**-2.28**	***Ccdc88a***	***girdin***	**-1.56**	***Mpp5***		**1.65**	***Gpr155***		**1.52**
***H2-Eb2***		**-2.45**	***Eps8l1***		**-1.57**	***Prrg4***		**2.42**	***Gsto1***		**1.63**
***H2-Ob***		**-2.17**	***Marcks***		**-2.24**	***Rttn***		**1.65**	***Ifit3***		**2.71**
***Procr***	***CD201***	**-1.51**	***Tns1***		**-1.57**	***Slc38a2***		**1.55**	***Inpp4b***		**1.66**
						***Slc44a1***		**5.21**	***Klhl7***		**1.66**
**Ig-domain or IG-like**			**Mitochondria**			***Tmco1***		**1.91**	***Lage3***		**1.61**
***Cd200r4***		**2.15**	***Bckdhb***		**1.90**	***Tmem87b***		**1.61**	***Lrrc40***		**1.65**
***Cd7***		**1.65**	***Cyba***		**1.73**	***Tmem167b***		**1.92**	***Mageh1***	***1-Apr***	**1.55**
***Gm13892***		**2.27**	***Fam136a***	***Hyccin***	**2.02**	***Tmem176b***		**1.80**	***Memo1***		**1.72**
***Treml2***		**1.66**	***Gcsh***		**2.20**	***Tmem203***		**1.75**	***Mipol1***		**1.75**
***Cd200***	***OX2***	**-2.59**	***Higd1a***		**1.53**	***Trdn***		**2.49**	***Nans***		**1.50**
***Fcrla***		**-1.65**	***Mut***		**1.61**	***1810006K21Rik***	***Tmem258***	**1.52**	***Nat2***		**1.79**
***Gm26***	***Trav4d-4***	**-1.79**	***Ndufa12***		**1.51**	***1810048J11Rik***	***Pcnxl4***	**1.50**	***Ncapg***		**1.83**
***Gm5571***	***Igkv9-120***	**-1.90**	***Ndufc2***		**1.61**	***3110001D03Rik***		**1.89**	***Nfkbib***	***Ikb***	**1.54**
***Igl-V1***		**-1.99**	***Parl***		**1.86**	***Cd180***	***Ly78***	**-2.54**	***Plac8***		**2.14**
***Igl-V2***		**-2.30**	***Prdx6***		**1.78**	***Kcnrg***		**-2.67**	***Plekha1***	***TAPP1***	**1.81**
***Siglecg***	***Siglec10***	**-1.67**	***Slc25a17***		**1.59**	***Mlec***		**-1.69**	***Prr13***		**1.65**
***Slamf1***	***CD150***	**-2.38**	***Tdrd7***		**1.60**	***Slc10a4***		**-1.56**	***Ptar1***		**1.54**
			***Timm17a***		**1.61**	***Slc12a6***		**-1.52**	***Pwp1***		**1.57**
**Complement and coagulation pathways**			***Uqcrq***		**1.59**	***Xkrx***		**-1.57**	***Qser1***		**1.89**
***Pros1***		**2.32**	***1110058L19Rik***		**1.57**				***Rg9mtd2***	***Trmt10a***	**1.60**
***C1qa***		**-1.56**	***2410091C18Rik***	***Ndufa7***	**1.53**	**Protein kinases**			***Rmrp***		**1.56**
***C1qc***		**-2.51**	***2810417H13Rik***		**2.20**	***Acvr2a***		**2.22**	***Rnu12***		**2.23**
***Cr2***	***CD21***	**-2.81**	***9030617O03Rik***		**1.82**	***Bmpr1a***		**3.86**	***Rnu3b1***		**1.59**
***Plaur***	***CD87***	**-1.66**	***Akr1b7***		**-1.61**	***Btk***		**1.56**	***Rrp15***		**1.61**
						***Cdk12***		**1.62**	***Samd3***		**1.97**
**Immune response**			**ATP metabolic process**			***Cdkl3***		**1.58**	***Scarna17***		**1.73**
***Tlr1***	***CD281***	**1.79**	***Atp5g2***		**1.60**	***Cpne3***		**1.61**	***Snhg1***		**3.36**
***Swap70***		**-2.24**	***Atpv0c***		**1.85**	***Mapk3***	***ERK1***	**1.54**	***Snora19***		**1.54**
			***Atp6v0d2***		**4.45**	***Mastl***		**1.65**	***Snord104***		**1.53**
**Killer-cell lectin-like receptors**			***Atp6v1e1***		**1.53**	***Prkag1***		**1.77**	***Snord118***		**1.87**
***Klra9***		**-1.73**	***Entpd1***	***CD39***	**1.59**	***Ryk***		**1.85**	***Snord15b***		**2.15**
***Klra10***		**-1.60**	***Fignl1***		**1.72**	***Yes1***		**2.18**	***Snord47***		**1.69**
***Klrb1c***	***NK1*.*1***	**-5.11**				***Irak3***		**-1.67**	***Spef2***		**1.73**
***Klrg1***		**-1.52**	**Oxidation reduction**			***Lrrk2***		**-2.65**	***Tatdn1***		**1.58**
			***Akr1c12***		**1.77**	***Lyn***		**-2.43**	***Tatdn2***		**1.56**
**Lectins**			***Akr1c13***		**2.06**	***Mapk11***	***P38b***	**-1.52**	***Tdp2***		**1.55**
***Clec2i***	***Clrg***	**1.57**	***Akr1e1***		**2.44**	***Sykb***		**-2.09**	***Ttc28***		**1.69**
***Fcer2a***	***CD23***	**-3.15**	***Hsd17b6***		**-1.71**				***Uprt***		**1.54**
***Mrc1***	***CD206***	**-2.11**				**Protein phosphatase**			***Vwa5a***		**1.62**
			**Lipid metabolism**			***Mtmr1***		**1.60**	***Wbp5***		**1.71**
**Nucleotide biosynthesis**			***Hsd17b12***		**1.84**	***Phpt1***		**2.06**	***Wdr45l***		**1.52**
***Nampt***	***Visfatin***	**2.30**	***Idi1***		**1.55**	***Ptpn12***		**1.52**	***Wdr61***		**1.61**
***Nme7***		**1.76**	***Mboat1***		**1.71**	***Styx***		**1.76**	***0610012G03Rik***		**1.85**
***Nt5e***	***CD73***	**1.77**	***Pdss1***		**1.79**				***1110059G10Rik***		**1.51**
***Rrm1***		**1.87**	***Ptdss1***		**1.62**	**G coupled protein receptor**			***1810020D17Rik***	***Aamdc***	**1.81**
						***Gpr63***		**1.70**	***2810026P18Rik***	***Snhg5***	**1.77**
**DNA repair**			**Fatty acid metabolism**			***Gpr114***		**1.75**	***4632404H12Rik***		**1.52**
***Hus1***		**1.62**	***Acadsb***		**2.12**	***Gpr174***		**1.57**	***4732471D19Rik***		**1.53**
***Nudt1***		**1.77**	***Adipor2***		**1.51**	***Dgkh***		**-1.87**	***4930422G04Rik***		**1.84**
***Alkbh3***		**-1.71**	***Elovl5***		**1.64**	***Emr4***	***Fire***	**-1.54**	***6330407A03Rik***		**1.51**
			***Elovl7***		**1.91**	***Gpr55***		**-1.63**	***9630013D21Rik***		**2.44**
**Apoptosis Regulation**			***Scd1***		**-4.13**	***Olfr98***		**-2.59**	***AY498738***	***Igkv9-129***	**-1.57**
***Casp4***		**1.69**				***Olfr164***		**-2.05**	***Bink***	***SLP65***	**-2.13**
***Chst11***		**1.53**	**Glucose metabolism**			***Olfr290***		**-2.57**	***Ccdc25***		**-1.71**
***Gpx1***		**1.52**	***Fn3k***		**1.77**	***Olfr707***		**-1.52**	***Gm10847***		**-2.84**
***Ngfrap1***		**1.74**	***Pdha1***		**1.67**	***Olfr745***		**-1.52**	***Gm4989***		**-1.64**
***Psmg2***	***Tnfsf5ip1***	**2.12**	***Pygl***		**2.48**	***Olfr1252***		**-1.67**	***Josd1***		**-1.92**
***Sap30bp***		**1.74**	***Gm9646***		**-1.57**				***LOC641050***		**-1.86**
***Apoe***		**-1.66**				**Peptidase inhibitor**			***LOC676175***		**-2.22**
						***Cst7***	***Cmap***	**1.66**	***Mir299***		**-1.50**
**Cell Death**						***Serpina3f***		**1.97**	***Rnf213***		**-1.80**
***Fgl2***		**1.95**				***2010005H15Rik***		**2.49**	***Speer4f***		**-1.50**
***Naip2***		**1.61**							***Stard10***		**-1.77**
***Naip5***		**2.23**				**GTPase regulator activity**			***Tmem22***		**-1.51**
***Rnf130***		**2.00**				***Asap2***		**1.58**	***Tox***		**-1.66**
***4930453N24Rik***	***Goliath***	**1.90**				***Myo9a***		**4.61**	***Wdr47***		**-1.64**
***Rnf216***		**-1.79**				***Arfgef2***		**-2.11**	***5730408K05Rik***		**-1.95**

Positive fold changes indicate increased expression in *late*M compared to *early*M CD62Lhi P14 cells

### Cytokine production, degranulation, and functional avidity of CD62Lhi memory CD8 T cells is not affected by time

**Fig 5:**
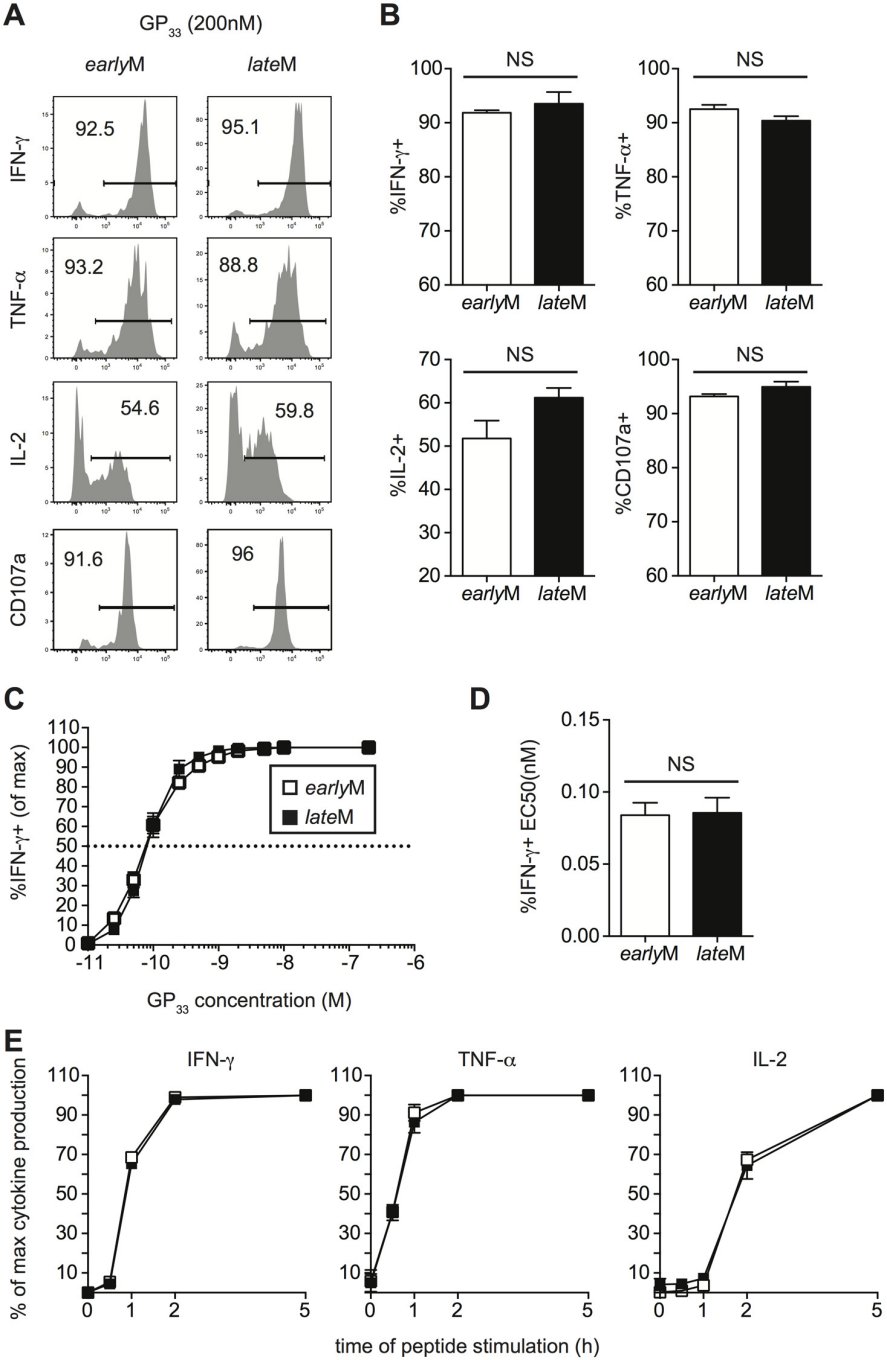
Cytokine production, degranulation, and functional avidity, of CD62Lhi memory are not influenced by time. Analysis was performed on CD62Lhi *early*M (30–45 days p.i.) and *late*M (8+ months p.i.) P14 cells. (A) Representative histograms from gated CD62L+ *early*M or *late*M P14 cells showing IFN-γ, TNF-α, or IL-2 production as measured by ICS, or surface CD107a expression as a measure of degranulation following 5hr incubation with GP_33-41_ peptide. (B) Percentage of gated CD62Lhi *early*M or *late*M P14 cells producing IFN-γ, TNF-α, or IL-2 or undergoing degranulation. (C) Functional avidity curves for CD62Lhi *early*M (open squares) and *late*M (black squares) P14 cells was determined by ICS following 5hr incubation of cells with the indicated concentrations of GP_33-41_ peptide. (D) EC50 for CD62Lhi *early*M and *late*M P14 cells as determined from functional avidity curves. (E) Time required for CD62Lhi *early*M (open squares) and *late*M (black squares) P14 cells to produce IFN-γ, TNF-α, or IL-2 was determined by ICS following incubation with GP_33-41_ peptide for the indicated lengths of time. NS, not statistically significant as determined by Student t-test. Representative data from one of three individual experiments with 3 mice per group per experiment. Error bars represent the standard error of the mean.

Memory CD8 T cells rapidly respond to infection with the production of cytokines and the release of cytolytic molecules including perforin and granzymes [3]. While microarray data did not indicate differences in mRNA expression of effector molecules between resting *early*M and *late*M P14 cells, this did not rule out the possibility that effector functions of CD62Lhi memory CD8 T cells change with time in response to Ag-stimulation. To examine if cytokine production by CD62Lhi memory CD8 T cells changes with time, Thy disparate *early*M and *late*M P14 cells were mixed together and incubated with GP_33-41_ peptide for 5 h followed by ICS for detection of IFN-γ, TNF-α, IL-2, and CD107a as a measure of degranulation. No differences in the production of any cytokines or degranulation were observed between *early*M and *late*M CD62Lhi P14 cells (Fig 5A and 5B).

To determine if sensitivity to Ag changes with time in CD62Lhi memory CD8 T cells, we performed ICS as described above using decreasing concentrations of GP_33-41_ peptide. No differences in Ag sensitivity were detected between CD62Lhi *early*M and *late*M P14 cells based upon functional avidity curves and the effective concentration of peptide required to induce 50% of cells to produce IFN-γ (EC50) (Fig 5C and 5D). Additionally, when *early*M and *late*M P14 cells were incubated with GP_33-41_ peptide for decreasing lengths of time, no differences in the time required to produce IFN-γ, TNF-α, or IL-2 were observed (Fig 5E), suggesting that there is a similarly poised state of *early*M and *late*M CD62Lhi to produce cytokines upon Ag recognition. As suggested from the microarray, these data indicate that effector functions of CD62Lhi memory CD8 T cells including cytokine production, secretion of cytolytic molecules, Ag sensitivity, and time required to produce cytokines does not change with time after infection.

To gain a broader appreciation for the poised state of *early*M compared to *late*M CD62Lhi cells, we incubated CD62Lhi *early*M and *late*M cells for 5 h in the presence or absence of GP_33-41_ peptide and examined expression of a number of effector molecules, surface markers, transcription factors, and cell cycle associated genes known to be dynamically regulated following Ag-encounter [41,48–50]. As we had previously noted by flow cytometry for expression of IFN-γ, TNF-α, and IL-2 (Fig 5A and 5B, S4A Fig), mRNA expression of effector molecules was similar between CD62Lhi *early*M and *late*M cells following incubation for 5 h with cognate Ag (S5A Fig). While expression of some genes including *Klrg1* and *Cx3Cr1* were differently expressed between resting CD62Lhi *early*M and *late*M cells (S5B Fig) as was indicated from the microarray data (Table 1), mRNA (S5B Fig) and surface protein (S4B Fig) expression of activation markers was similar for CD62Lhi *early*M and *late*M cells following the 5 h incubation period, indicating that CD62Lhi *early*M and *late*M cells are similarly activated following pathogen re-encounter. Additionally, mRNA (S5C Fig) and protein levels (S4C Fig) of transcription factors were regulated to a similar extent in CD62Lhi *early*M and *late*M cells following 5 h peptide incubation. Interestingly, while the microarray data indicated that CD62Lhi *late*M cells regulate expression of cell cycle related genes differently compared to *early*M cells (Fig 4B and Table 1), mRNA levels of cyclins and cyclin dependent kinases was simarly regulated in CD62Lhi *early*M and *late*M cells following 5 h incubation with cognate Ag (S5D Fig). Taken together, these data indicate that CD62Lhi *early*M and *late*M cells display a similarly poised state to respond following Ag re-encounter.

### Ability to undergo homeostatic and Ag-driven proliferation increases with time among CD62Lhi memory CD8 T cells

While mRNA expression of cell cycle genes following 5 h incubation with Ag indicated that genes regulating cell cycling were similarly regulated in CD62Lhi *early*M and *late*M cells following Ag re-encounter (S5D Fig), KEGG pathway analysis indicated that genes regulating cell cycle pathways are differentially expressed in CD62Lhi memory CD8 T cells with time (Fig 4B and S6A Fig). Additionally, gene set enrichment analysis (GSEA) [51] comparing gene expression patterns of CD62Lhi *early*M and *late*M P14 cells with existing gene sets revealed that genes highly expressed by CD62Lhi *late*M P14 cells were enriched in gene sets involved in cell cycle pathways (S6B Fig). Furthermore, while proliferation of CD62Lhi *early*M and *late*M cells incubated with cognate Ag *in vitro* was similar after 5 h, a greater percentage of CD62Lhi lateM compared to earlyM cells proliferated following incubation with cognate Ag for 24 h (S4D Fig). Taken together, these data suggested that CD62Lhi *late*M CD8 T cells might possess enhanced abilities to undergo homeostatic proliferation and/or Ag-driven proliferative expansion following re-infection.

**Fig 6:**
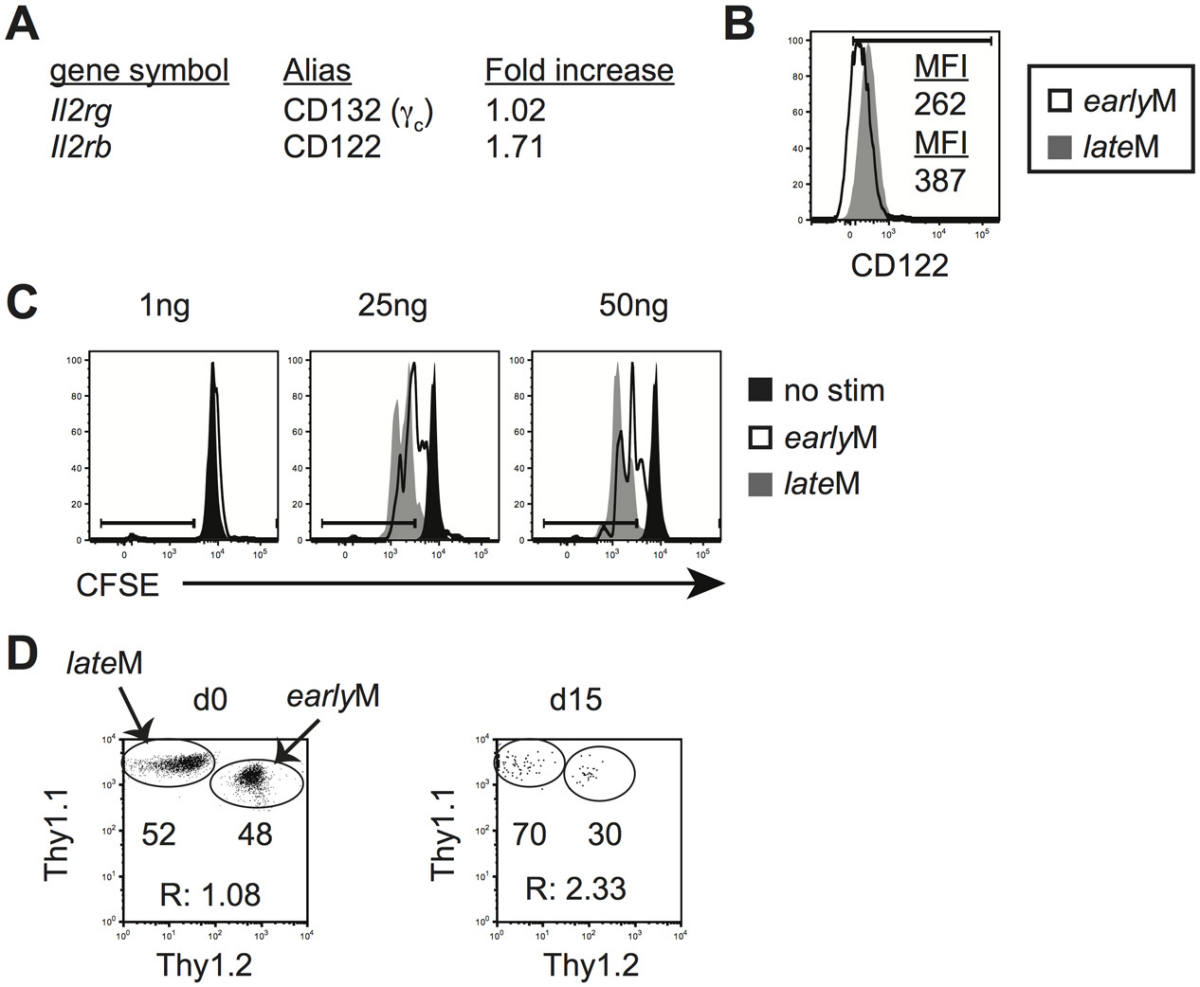
Sensitivity to IL-15 and ability to undergo homeostatic proliferation for CD62Lhi memory changes with time. Analysis was performed on CD62Lhi *early*M (30–45 days p.i.) and *late*M (8+ months p.i.) P14 cells. (A) A comparison of relative mRNA expression of the indicated components of the IL-15R complex between CD62Lhi *early*M and *late*M P14 cells from microarray data. Positive fold changes represent genes with increased expression in CD62Lhi *late*M compared to *early*M P14 cells. (B) Representative histograms of CD122 expression on gated CD62Lhi *early*M (open histogram) and *late*M (grey histogram) P14 cells. (C) Representative histograms of CFSE dilution on gated CD62Lhi *early*M (open histograms) or *late*M (grey histograms) P14 cells after 3 day culture in the presence or absence (black histograms) of the indicated concentrations of IL-15. (D) CD62Lhi *early*M and *late*M P14 cells were sorted and co-transferred into Rag-/- mice. *Left*. Dot plot showing the master mix of *early*M (Thy1.1/1.2) and *late*M (Thy1.1/1.1) P14 cells used for adoptive transfer. *Right*. Representative dot plot of transferred CD62Lhi *early*M (Thy1.1/1.2) and *late*M (Thy1.1/1.1) P14 cells isolated from spleens of Rag-/- mice d15 after adoptive transfer. R:, ratio of CD62Lhi *late*M to *early*M P14 cells. **, statistically significant (p<0.01) as determined by Student t-test.

Basal and homeostatic proliferation is partially dependent upon IL-15 [33–35]. IL-15 bound to IL-15Rα is trans-presented to CD8 T cells and signals through the common receptor γ chain (γ_c_, CD132) and IL-2Rβ (CD122) [52]. mRNA expression of CD122 as determined from microarray data (Fig 6A) and surface expression of CD122 as detected by flow cytometry (Figs 3A and 6B), increased with time in CD62Lhi memory P14 cells, suggesting that sensitivity to IL-15 could increase in CD62Lhi memory CD8 T cells with time after infection. To test this, Thy disparate *early*M and *late*M P14 cells were labeled with CFSE, mixed and incubated with increasing concentrations of IL-15, and dilution of CFSE was determined after 3 d. CD62Lhi *late*M P14 cells proliferated to a greater extent in response to exogenous IL-15, and CD62Lhi *late*M cells were more sensitive to lower levels of IL-15 compared to CD62Lhi *early*M P14 cells (Fig 6C). Endogenous GP_33_ and GP_276_ CD62Lhi *late*M cells also displayed increased expression of CD122 compared to *early*M cells (S1B Fig) suggesting they possesses increased sensitivity to IL-15 compared to *early*M CD62Lhi cells. Taken together, this data indicates that sensitivity to IL-15 increases over time in CD62Lhi memory CD8 T cells.

Differences in sensitivity to IL-15 suggested that CD62Lhi *early*M and *late*M CD8 T cells could differ in their ability to undergo homeostatic proliferation. To test this, Thy disparate CD62Lhi *early*M and *late*M P14 cells were sorted, mixed in equal numbers (Fig 6D left panel), injected into lymphopenic Rag-/- mice (3x10^4^ each), and the percentage of CD62Lhi *early*M and *late*M cells was determined in the spleens of recipients 15 d after transfer. A higher percentage of *late*M cells was found with the ratio of *late*M to *early*M cells increasing approximately two-fold (Fig 6D right panel), indicating that ability to undergo homeostatic proliferation increases with time in CD62Lhi memory CD8 T cells.

**Fig 7:**
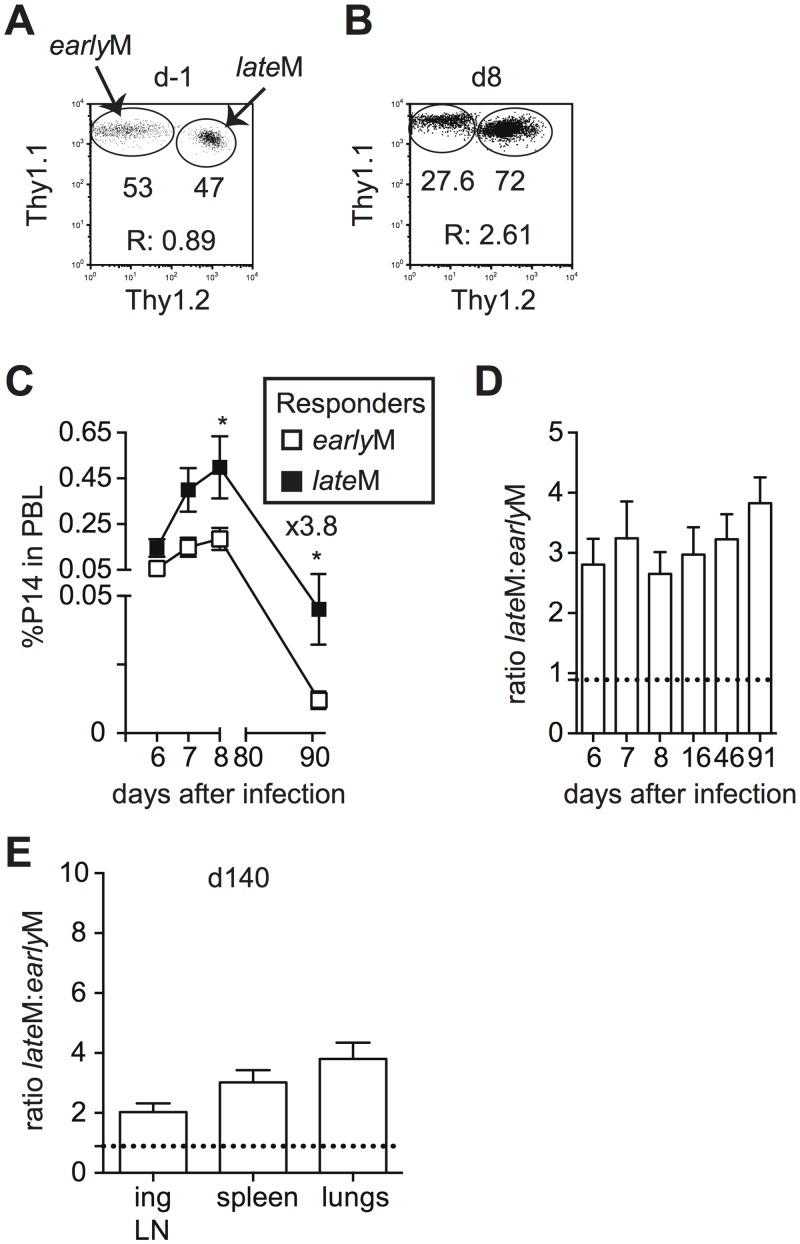
Proliferation and ‘memory generation potential’ of CD62Lhi memory cells increases with time after infection. Sorted CD62Lhi Thy disparate *early*M and *late*M P14 cells were mixed and injected into naïve recipients followed by i.p. injection of LCMV Armstrong 24 hours later. (A) Dot plot showing the master mix of CD62Lhi *early*M (Thy1.1/1.1) and *late*M (Thy1.1/1.2) P14 cells used for adoptive transfer. (B) Representative plot showing the response of progeny of sorted CD62Lhi *early*M (Thy1.1/1.1) and *late*M (Thy1.1/1.2) P14 cells in PBL d8 after LCMV infection. (C) Kinetic analysis of secondary responses generated from sorted CD62Lhi *early*M (open squares) and *late*M (black squares) P14 cells in PBL at the indicated days post LCMV infection. (D) Ratio of cells generated from sorted CD62Lhi *late*M to *early*M P14 cells in PBL at the indicated days post LCMV infection. Doted line indicates the starting ratio of CD62Lhi *late*M to *early*M P14 cells. (E) Ratio of cells generated from sorted CD62Lhi *late*M to *early*M P14 cells in the indicated organs at a memory time point (d140) following LCMV infection. Doted line indicates the starting ratio of CD62Lhi *late*M to *early*M P14 cells. R:, ratio of CD62Lhi *late*M to *early*M P14 cells or progeny of CD62Lhi *late*M to *early*M P14 cells.*, statistically significant (p<0.05) as determined by Student t-test. Representative data from one of two individual experiments with 5 mice per group per experiment. Error bars represent the standard error of the mean.

Differences in cell cycle regulation also could indicate that the ability to undergo Ag-driven proliferation changes with time among CD62Lhi memory CD8 T cells. To test this, Thy disparate CD62Lhi *early*M and *late*M P14 cells were sorted, mixed in equal numbers (Fig 7A), and 1x10^4^ of each were transferred into naïve C57BL/6 recipients followed by infection with LCMV 24 h later. An increased percentage of progeny were generated from CD62Lhi *late*M P14 cells during the effector phase (Fig 7B–7D), indicating that with time CD62Lhi memory CD8 T cells have an increased ability to undergo secondary expansion. Adoptive transfer of endogenous CD62Lhi GP_276_-specific *early*M and *late*M cells (S7A Fig) also showed increased numbers of 2° effector cells generated from *late*M cells compared to *early*M cells in PBL and spleen (S7B and S7C Fig), and nearly 4 times the number of 2° effector cells generated from CD62Lhi *late*M cells compared to *early*M cells were recovered in spleens 7 days post LCMV infection (S7C Fig). Additionally, when progeny generated from CD62Lhi *early*M and *late*M P14 cells were examined at a memory time point in PBL (Fig 7C and 7D) or in peripheral tissues and secondary lymphoid organs (Fig 7E), a greater percentage of secondary memory cells were generated from CD62Lhi *late*M compared to *early*M, indicating that with time, memory generation potential increases for CD62Lhi memory CD8 T cells. As suggested by the microarray data, these results indicate that with time after infection, the ability to undergo Ag-driven proliferation and generate secondary memory cells increases within CD62Lhi memory CD8 T cells.

### Protective capacity of memory CD8 T cells changes with time in a pathogen dependent manner

**Fig 8:**
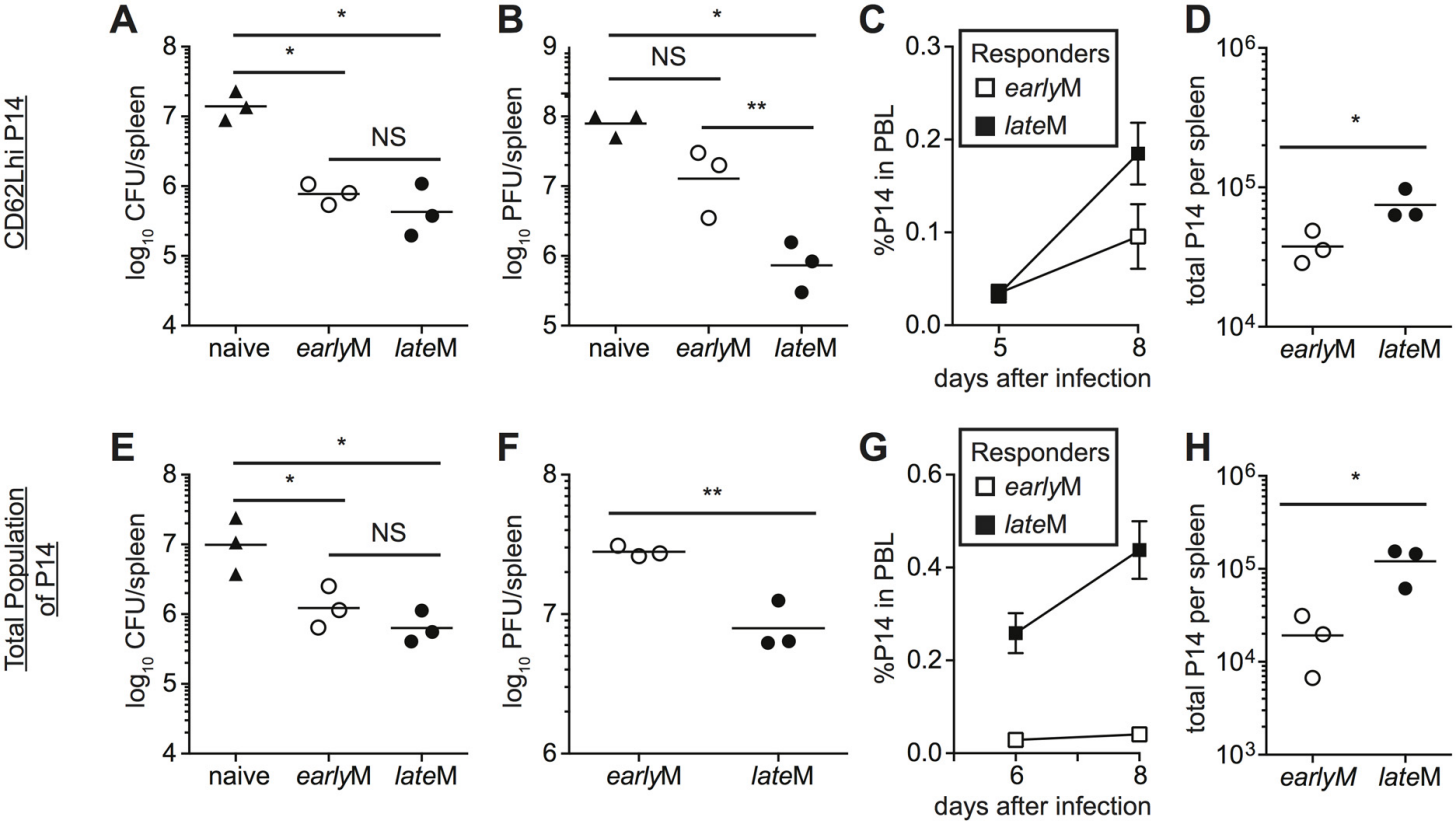
*late*M cells provide better protection than *early*M cells following LCMV clone-13 infection. (A-D) Sorted CD62Lhi *early*M (30–45 days p.i.) or *late*M (8+ months p.i.) P14 cells were injected into naïve recipients followed by i.v. injection of LM or LCMV clone-13 24 hours later. (E-F) Positively selected *early*M (30–45 days p.i.) or *late*M (8+ months p.i.) populations of P14 cells were injected into naïve recipients followed by i.v. injection of LM or LCMV clone-13 24 hours later. (A and E) Bacterial CFUs in the spleen were measured three days after LM infection for mice that received no cells (naïve), or adoptive transfer of 7x10^4^ sorted CD62Lhi *early*M or *late*M P14 cells (A) or 7x10^4^ positively selected *early*M or *late*M P14 cell populations (E). (B and F) Viral PFUs in the spleen were measured 8 days after LCMV clone-13 infection for mice that received no cells (naïve), or adoptive transfer of 5x10^4^ sorted CD62Lhi *early*M or *late*M P14 cells (B) or 7x10^4^ positively selected *early*M or *late*M P14 cell populations (F). (C and G) Kinetic analysis of secondary responses generated from sorted CD62Lhi *early*M (open squares) or *late*M (black squares) P14 cells (C) and secondary responses generated from positively selected *early*M (open squares) or *late*M (black squares) P14 cell populations (G) in PBL at the indicated days post LCMV clone-13 infection. (D and H) Numbers of progeny P14 cells generated from transferred CD62Lhi *early*M or *late*M cells (D) and transferred *early*M or *late*M cell populations (H) found in the spleens of recipient mice 8 days post LCMV clone-13 infection. NS, not statistically significant; *, statistically significant (p<0.05) as determined by Student t-test or ANOVA with a Bonferroni post-test. Representative data from one of two to three individual experiments with 3 mice per group per experiment. Error bars represent the standard error of the mean.

The goal of vaccination is establishment of memory populations that will provide increased protection upon infection, and studies have indicated that the quantity, quality, and localization of memory CD8 T cells required for protection differ depending upon the nature of the pathogen [6,8–10,53,54]. The functional differences that we observed suggested that CD62Lhi *early*M and *late*M cells might provide differing levels of protection following infection. To determine if per cell protective capacity of CD62Lhi memory CD8 T cells against an acute systemic infection changes with time, CD62Lhi *early*M and *late*M P14 cells were sorted, and 7x10^4^ cells were transferred into naïve C57BL/6 recipients followed by infection with virulent LM expressing GP_33_. Both CD62Lhi *early*M and *late*M P14 cells provided protection, as significantly decreased (p<0.05) colony forming units (CFUs) of LM were detected in spleens of recipient mice three days after infection compared to mice not receiving adoptive transfer. However, per cell protective capacity of CD62Lhi *early*M and *late*M P14 cells did not differ (Fig 8A). The most dramatic alteration in functional ability between CD62Lhi *early*M and *late*M CD8 T cells that we observed was the ability to undergo Ag-driven proliferation, and some studies have indicated that the proliferative abilities of memory CD8 T cells are less important than localization and killing ability for providing protection from LM [8–10]. However, studies have shown that proliferative abilities of memory CD8 T cells are crucial for clearance of infection with LCMV clone-13, which causes a chronic infection in mice [6,10,54]. To determine if per cell protective capacity of CD62Lhi memory CD8 T cells against a chronic infection changes with time, we sorted CD62Lhi *early*M and *late*M P14 cells and transferred 5x10^4^ cells into naïve C57BL/6 recipients followed by infection with LCMV clone-13. Mice that received adoptive transfer of CD62Lhi *late*M cells had reduced viral titers eight days following infection compared to mice not receiving transferred cells, and this level of protection was significantly greater than that provided by CD62Lhi *early*M cells (Fig 8B). Increased protection provided by *late*M CD62Lhi cells correlated with enhanced magnitudes of proliferative expansion as a greater percentage of progeny generated from CD62Lhi *late*M compared to *early*M cells was found in the PBL (Fig 8C), and higher numbers of progeny generated from CD62Lhi *late*M were found in the spleens of recipient mice 8 days p.i. (Fig 8D). Taken together, these data indicate that protection provided by CD62Lhi memory CD8 T cells changes with time after infection in a pathogen-dependent manner.

Protection provided against infection is mediated by all memory cells present at the time of re-infection. When analyzed on the population level (i.e. no sorting based on CD62L expression), *late*M CD8 T cells proliferated to a greater extent following acute infection compared to *early*M populations (Fig 2B), suggesting that protection provided by populations of memory CD8 T cells may also differ with time. To determine if per cell protective capacity of memory CD8 T cell populations changes with time, cells were isolated from the spleens of mice containing *early*M or *late*M P14 cells, and 7x10^4^ cells were transferred into naïve C57BL/6 recipients followed by infection with virulent LM expressing GP_33_. Both *early*M and *late*M populations provided protection against LM, but no difference in the level of protection was observed (Fig 8E). To determine if protection provided by memory CD8 T cell populations against a chronic infection changes with time after infection, 5x10^4^ *early*M or *late*M cells were transferred into naïve C57BL/6 recipients followed by infection with LCMV clone-13. *late*M populations provided enhanced protection against chronic LCMV clone-13 infection compared to *early*M populations (Fig 8F). Enhanced protection provided by *late*M CD8 T cells also correlated with enhanced secondary expansion in PBL and greater numbers of P14 cells recovered from spleens of infected mice 8 days following infection (Fig 8G and 8H). Taken together, these data indicate that the ability of memory CD8 T cells to provide protection against chronic infection increases with time after infection in a manner not due solely to shifts in T_em_ to T_cm_ subsets.

### Mitochondrial function of memory CD8 T cells improves with time after infection

**Fig 9:**
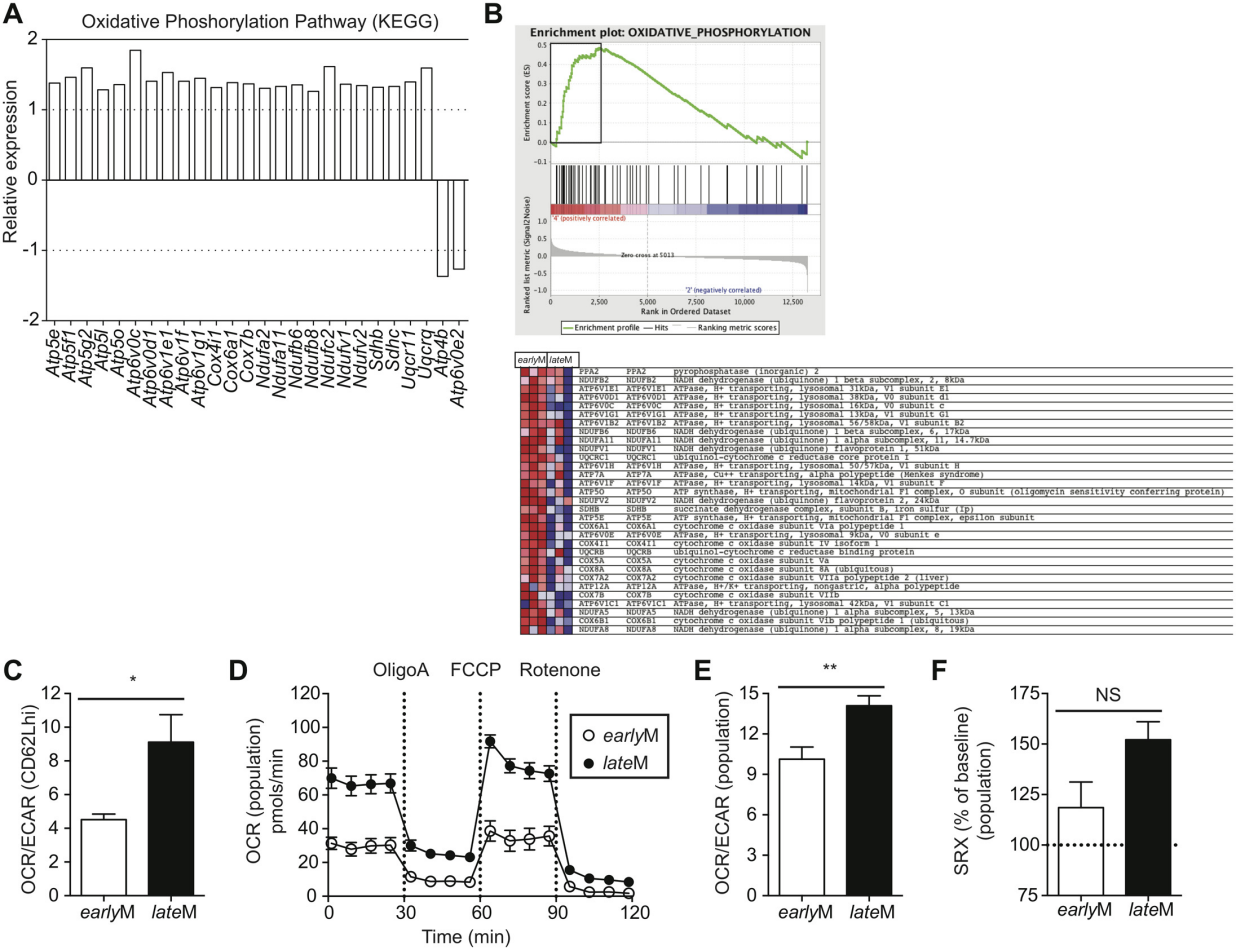
Mitochondrial function of memory CD8 T cells increases with time after infection. (A) Biological pathway analysis based on microarray data of genes with significant mRNA changes of fold >1.25 in CD62Lhi *early*M and *late*M P14 cells was generated using the KEGG pathway tool in DAVID bioinformatics resources. Shown is relative gene expression for genes involved in the KEGG oxidative phosphorylation pathway that are differentially expressed between CD62Lhi *early*M and *late*M P14 cells. Positive fold changes represent genes with increased expression in CD62Lhi *late*M compared to *early*M P14 cells while negative fold changes represent genes with decreased expression in CD62Lhi *late*M compared to *early*M P14 cells. (B) Gene set enrichment analysis was performed comparing expression of genes in CD62Lhi *early*M and *late*M P14 cells to existing gene sets. *late*M cells showed enrichment for oxidative phosphorylation gene sets. (C-F) *early*M (30–45 days p.i.) and *late*M (8+ months p.i.) populations or CD62Lhi P14 cells were purified and 2x10^5^ cells were plated in XF media and analyzed with an XF-96 extracellular flux analyzer (Seahorse Bioscience). (C) Ratio of O_2_ consumption rates (OCR) to extracellular acidification rates (ECAR) measured under basal conditions for CD62Lhi *early*M or *late*M P14 cells. (D) OCR was measured under basal conditions and in response to the indicated mitochondrial inhibitors for *early*M or *late*M P14 cell populations. (E) Ratio of OCR to ECAR measured under basal conditions for *early*M or *late*M P14 cell populations. (F) Spare respiratory capacity (SRC) of *early*M or *late*M P14 cell populations as indicated by maximum OCR following FCCP injection as a percentage of maximum OCR under basal conditions. NS, not statistically significant; *, statistically significant (p<0.05); **, statistically significant (p<0.01) as determined by Student t-test. Combined data from four individual experiments that provided similar results with 3 mice per group per experiment. Error bars represent the standard error of the mean.

Alterations in metabolic function including enhanced fatty acid oxidation, increased mitochondrial mass, and increased ability to perform oxidative phosphorylation have been shown to enhance memory CD8 T cell development and favor rapid recall responses following Ag re-encounter [44–47,55]. Additionally, memory CD8 T cells that displayed an enhanced ability to undergo oxidative phosphorylation and that possessed increased spare respiratory capacity (SRC), which is the reserve ATP generation capacity of cellular mitochondria, proliferated to a greater extent and provided enhanced protection against chronic infection with LCMV clone-13 [54]. Functional annotation and KEGG pathway analysis of our microarray data revealed that genes regulating mitochondrial function and metabolic pathways including oxidative phosphorylation; fructose, mannose, and glucose metabolism; and fatty acid oxidation were differently regulated between CD62Lhi *early*M and *late*M CD8 T cells (Table 1, Figs 4B and 9A). Additionally, GSEA analysis revealed that *late*M CD62Lhi CD8 T cells were enriched in genes sets involved in oxidative phosphorylation (Fig 9B). This suggested that CD62Lhi memory CD8 T cells might alter their metabolic programs with time after infection, which would impact their ability to proliferate and to provide protection against LCMV clone-13 infection.

To determine if the metabolic function of CD62Lhi memory CD8 T cells is altered with time after infection, we sorted *early*M and *late*M CD62Lhi memory CD8 T cells and performed extracellular flux analysis using a Seahorse bioanalyzer. Comparison of the basal oxygen consumption rate (OCR), a measure of oxidative phosphorylation, to the extracellular acidification rate (ECAR), a measure of aerobic glycolysis, revealed that compared to CD62Lhi *early*M cells, CD62Lhi *late*M cells rely to a greater extent on oxidative phosphorylation for the generation of ATP (Fig 9C). Due to the demands of cell sorting, we were unable to determine SRC of CD62Lhi *early*M and *late*M cells, as they did not respond to the fluorocarbonyl cyanide phenylhydrazone (FCCP) inhibitor. However, *late*M CD8 T cell populations also proliferated to a greater extent than *early*M populations and provided enhanced protection against LCMV clone-13, and these populations could be isolated without using cell sorting. To examine if the metabolic function of CD8 T cell memory populations is altered with time after infection we performed extracellular flux analysis of isolated *early*M and *late*M CD8 T cell populations. *late*M cells displayed increased basal OCR levels compared to *early*M cells, and both cell types responded to metabolic inhibitors (Fig 9D). Comparing the ratio of OCR to ECAR for *early*M and *late*M populations reveled that *late*M cells rely more heavily on oxidative phosphorylation for ATP production compared to *early*M populations (Fig 9E). Analysis of the SRC, calculated as the highest OCR after addition of FCCP over the basal OCR, for *early*M and *late*M revealed a trend for higher SRC in populations of *late*M cells compared to *early*M cells (Fig 9F). Taken together, these data indicate that mitochondrial function of memory CD8 T cells improves with time after infection in a manner that is not solely due to shifts in T_cm_ and T_em_ subsets. The enhanced mitochondrial function of *late*M populations and CD62Lhi cells likely provides *late*M cells with a metabolic advantage enabling robust proliferation and enhanced protection against chronic infection with LCMV clone-13.

## Discussion

Protection provided by memory CD8 T cells is dependent upon their numbers, functional ability (quality), and location at the time of infection [56]. We have shown that the quality of the circulating memory CD8 T cell population differs with time after infection in a manner not solely due to shifts in memory subset composition. Some functions of memory CD8 T cells analyzed on the population level, such as ability to produce IL-2, increased with time after infection, but were no different in CD62Lhi memory cells early or late after infection. However, other qualitative aspects of memory CD8 T cells including proliferation in response to the homeostatic cytokine IL-15 or to Ag, and mitochondrial function increased with time after infection when both the memory CD8 T cell population, and defined CD62Lhi subsets were analyzed. Thus, while some alterations in the functional abilities of memory CD8 T cells with time after infection can be attributed to shifts in subset composition, other qualitative changes cannot be wholly attributed to shifts in subset composition. Interestingly, as a consequence of these functional changes, the protection provided by memory CD8 T cell populations and CD62Lhi memory CD8 T cells against a chronic viral infection increased over time. Importantly, our data suggests that the outcome vaccination schemes designed to elicit protective memory CD8 T cells will depend on the timing between booster immunizations, and on the timing of re-infection following vaccination.

While circulating memory CD8 T cells are best suited to provide protection against systemic infections, tissue resident memory CD8 T cells provide a first line of defense against pathogens encountered in peripheral tissues [7,11–15]. While the longevity of T_rm_ cells relative to circulating memory CD8 T cells is unclear at present, some studies have indicated that T_rm_ cells remain in mice for at least 300 days following infection with vaccinia virus [12], herpes virus [15], or vesicular stomatitis virus [57]. However, other studies examining T_rm_ formation following infection with influenza virus have indicated that the T_rm_ CD8 T cell population wanes following infection [17]. Thus, longevity of T_rm_ CD8 T cells may vary depending on factors including the nature of the primary infection and/or vaccination and the tissue of residence. Additionally, studies examining the longevity and phenotypic and functional changes that might occur in T_rm_ CD8 T cells over time following infection will be complicated due to a lack of phenotypic markers that definitively identify T_rm_ cells, as CD103 and CD69, markers used to identify T_rm_ cells, are not expressed on all T_rm_ cells [7,58,59]. However, T_rm_ CD8 T cells likely would play an important role in providing protection against human pathogens that infect at peripheral tissues, including HIV, herpes viruses, and influenza viruses. Therefore, determining the longevity of Trm cells and whether the phenotype, function, and protective abilities of Trm cells differs with time after infection, as we have shown for circulating memory CD8 T cells, is an important goal.

While we have provided evidence that changes in memory CD8 T cell phenotype and function seen on the population level are not due solely to conversion to CD62Lhi cells with time after infection, a question still remains as to how the progressive changes in phenotype and function seen with time after infection in both memory CD8 T cell populations and within CD62Lhi memory CD8 T cells occurs. Our microarray data suggests that transcription of genes important for memory CD8 T cell function are differentially regulated in CD62Lhi memory CD8 T cells with time after infection, but it is unclear if differences in the transcriptional program with time after infection are due to 1) cell-intrinsic changes in gene regulation, or 2) a subset of memory CD8 T cells within the *early*M population selectively survives and comes to constitute the *late*M pool. To ideally address these possibilities, *early*M CD8 T cell subsets would be transferred into naïve mice, and the phenotype, function, protective abilities, and transcriptional regulation of the transferred cells would be analyzed for the *late*M cells derived from the transferred populations. However, due to the low number of CD62Lhi memory CD8 T cells present early following infection, loss of cells upon adoptive transfer, and further loss of cells upon isolation from recipient mice, these experiments are difficult to execute. We hope that improvements in cell isolation technology will allow us to perform these experiments in the future. We considered the possibility that transcription factors that facilitate memory CD8 T cell formation such as Tbet, Eomes, or Tcf1 [60–62] could differentially regulate survival of subsets of memory cells displaying phenotypic markers that were present in *early*M cells but not present in *late*M cells as seen in S4 Fig. However, while expression of Eomes and Tcf1 differed between *early*M and *late*M populations (S8 Fig), we were unable to find conclusive evidence that expression of these transcription factors regulated survival of subsets within CD62Lhi *early*M and *late*M cells.

The rate at which phenotypic and functional changes of memory CD8 T cells occur following infection and/or vaccination is likely to be influenced by a number of factors. In this study we examined Tg memory P14 cells primarily localized within the spleen following acute infection with LCMV. Previous studies have indicated that the rate of acquisition of effector functions and expression of surface markers associated with central memory CD8 T cells is influenced by the number of transferred Tg T cells [24,63,64]. However, expression of CD62L following adoptive transfer of low numbers of Tg P14 cells as used in our study has been shown to be similar between Tg and endogenous D^b^-gp33 restricted CD8 T cells [24,63]. On the other hand, the rate of surface CD62L expression and ability to produce IL-2 has been shown to differ between endogenous T cell populations of different LCMV epitope specificities and among CD8 T cells localized within secondary lymphoid organs or peripheral tissues [24]. Additionally, the nature of the infecting pathogen and/or inflammation elicited during either infection or vaccination has been shown to influence the phenotype and function of memory CD8 T cells generated during the response. Infection with LCMV, vaccinia virus, or influenza virus leads to the formation of memory CD8 T cells with distinct phenotypic and functional qualities [65,66], while administration of inflammation inducing toll-like receptor agonists during dendritic cell (DC) immunizations abrogates the rapid acquisition of memory characteristics seen during low-inflammatory DC immunizations [67–69]. Because of these considerations, the extent of changes that occur within the CD8 T cell memory population with time, and thus their functional and protective abilities during re-infection will likely depend upon conditions elicited during the primary infection and/or immunization. Therefore, vaccine design should include considerations of how the vaccine strategy may influence changes in memory CD8 T cells with time.

Unlike mice housed in specific pathogen free facilities, humans are infected with many non-related pathogens, and co-infections or chronic infections could influence the development and/or differentiation of primary memory CD8 T cells, or the properties of already established memory CD8 T cells. A recent report showed that established chronic infections in mice influence the development and differentiation of primary memory CD8 T cells, but that the impact of chronic infections on pre-established primary memory CD8 T cells was less severe [70]. However, pre-established memory CD8 T cells examined in their study were generated 1 year prior to the chronic infection. It will be interesting to examine if differentiation of more recently established memory CD8 T cells is also minimally impacted by chronic or repeated unrelated infections.

Studies have indicated that the quantity, quality, and localization of memory CD8 T cells required for protection differ depending upon the nature of the pathogen [6,8–10,53,54]. We found that the protective abilities of memory CD8 T cells changes with time in a pathogen-dependent manner. CD62Lhi central memory cells have been described as being more effective at providing protection against chronic infections due to localization within the lymph nodes and increased mitochondrial function leading to an enhanced ability to proliferate [6,10,54]. We showed that the ability of memory CD8 T cell populations and CD62Lhi central memory CD8 T cells to provide protection from LCMV clone-13 infection increases with time, and that this increased protection correlated with enhanced mitochondrial function and proliferative abilities following infection. Some studies [8–10], however, have indicated that memory CD8 T cells with effector memory characteristics provide increased protection against acute infection with *L*. *monocytogenes* or localized infection with vaccinia virus, and the localization of the memory population to sites of infection is important in these instances. Therefore, increased protection provided by *late*M cells likely does not apply to all infections.

The numbers of memory CD8 T cells required to achieve protection against certain pathogens including *Plasmodium* species which cause malaria is quite high, and prime boost protocols have been established in order to achieve high numbers of memory CD8 T cells [29–31]. Our data indicate that higher numbers of memory CD8 T cells may be achieved through prime boost protocols by increasing the length of time between boosts. However, our study analyzed primary memory cells, and recent studies have indicated that the properties of memory CD8 T cells including magnitude of proliferative expansion, duration and degree of contraction, cytotoxicity, IL-2 production, basal proliferation and long-term survival, memory generation potential, lymph node homing, and transcriptome diversification change sequentially with each additional Ag encounter [71–73]. While little is known about how the number of Ag encounters influences the changes in memory CD8 T cell functions that occur with time after infection, studies indicate that the phenotype of memory CD8 T cells that have encountered Ag multiple times changes with time after infection, but at a slower rate than in primary memory CD8 T cells [25,28]. As with primary memory, changes with time in the properties of memory CD8 T cells that have encountered Ag more than once could influence their ability to provide protection against infection and/or affect the outcome of prime boost immunizations requiring multiple boosts.

Our results firmly establish that memory CD8 T cells continue to change with time after infection. The results indicate that the function of memory CD8 T cells continues to change with time after infection, and that protection provided by memory CD8 T cells changes with time in a pathogen-dependent manner. Because of this, experimental investigation of memory CD8 T cell quality and/or protection following either infection or vaccination should include analysis of memory CD8 T cells at multiple time points following the infection and/or vaccination.

## Materials and Methods

### Ethics statement

All experimental procedures utilizing mice were approved by the University of Iowa Animal Care and Use Committee under the ACURF protocol number 1202050. The experiments performed in this study were done under strict accordance to the Office of Laboratory Animal Welfare guidelines and the PHS Policy on Humane Care and Use of Laboratory Animals.

### Mice, bacteria, and viruses

C57BL/6 Thy1.2 mice were obtained from the National Cancer Institute (Frederick, MD). B6/SJL (CD45.1), Rag-/- mice, and P14 mice were bred at the University of Iowa (Iowa City, IA). The Armstrong strain of LCMV, the clone-13 strain of LCMV, Vaccinia Virus expressing the GP33 epitope (VacV), attenuated *actA-* deficient *Listeria monocytogenes* expressing the GP33 epitope [31], and virulent *Listeria monocytogenes* strain 1043S expressing the GP33 epitope were grown and quantified as previously described [10,27].

### Adoptive transfers and generation of *early*M and *late*M P14 and endogenous memory cells

For generation of *early*M and *late*M P14 cells, P14 CD8 T cells (specific for the LCMV GP_33-41_ epitope) were isolated from the peripheral blood of young Thy1.1/1.1 or Thy1.1/1.2 P14 mice. Contaminating memory phenotype (CD11a^hi^/CD44^hi^) P14 cells were always <5%. 5x10^3^ P14 cells were transferred retro-orbitally into 6–12 week old naïve C57BL/6 mice, and recipients were infected 24 h later intraperitoneally (i.p.) with 2x10^5^ plaque forming units (PFU) of LCMV Armstrong (LCMV). All *early*M analysis was done between 30–45 days after infection and *late*M analysis was done 8+ months after infection. For co-transfer of *early*M and *late*M P14 cells, P14 cells were isolated from the spleens of mice containing Thy disparate *early*M and *late*M P14 cells, mixed at a 1:1 ratio, and 2x10^4^ of each were transferred retro-orbitally into naïve C57BL/6 mice followed 24 hours later by i.p. injection of 2x10^5^ PFU of LCMV or 3x10^6^ PFU of VacV, or by intravenous (i.v.) injection of 5x10^6^ colony forming units (CFUs) of LM. For co-transfer of CD62Lhi *early*M and *late*M P14 cells, P14 cells were isolated from the spleens of mice containing Thy disparate *early*M and *late*M P14 cells, and cells were surface stained for Thy1.1, CD8, and CD62L (eBioscience) and sorted using a BD FACSAria II (BD Biosciences). Sorted cells were mixed at a 1:1 ratio and 1x10^4^ of each were transferred retro-orbitally into naïve C57BL/6 mice followed 24 hours later by i.p. injection of 2x10^5^ PFU of LCMV. Sorted cells were >95% pure. Input ratios of *early*M and *late*M P14 cells were confirmed by flow cytometry before adoptive transfer. For adoptive transfer of CD62Lhi endogenous GP_276_ cells, splenocytes of *early*M and *late*M C57BL/6 (CD45.2) mice were stained with PE-anti-CD8 antibodies and purified with anti-PE magnetic bead sorting using standard AutoMacs protocols. Cells were then stained with CD62L, and CD62Lhi cells were sorted using a BD FACSAria II (BD Biosciences). Following sorting, cells were stained with GP_276_ tetramer to determine the percentage of endogenous GP_276_ memory cells in the sorted CD62Lhi CD8 T cell population. 2.5x10^3^ endogenous CD62Lhi *early*M or *late*M GP_276_ tetramer positive cells were then transferred into CD45.1 C57/SJL mice followed 24 hours later by i.p. injection of 2x10^5^ PFU of LCMV.

### Lymphocyte isolation, quantification of CD8 T cell responses and surface marker expression, ICS and degranulation analysis, transcription factor staining, and KI67 staining

*early*M and *late*M CD8 T cell responses were quantified in peripheral blood by collecting blood via retro-orbital puncture. Red blood cells were lysed with ACK, and P14 cells were surface stained for Thy1.1, Thy1.2, and CD8 (eBioscience), and endogenous memory cells were surface stained for Thy1.1, CD45.2, CD8 (eBioscience), and GP_276_ tetramer. Cells were acquired on a FACSCalibur instrument (BD Biosciences), and Thy expression was used to distinguish between *early*M and *late*M P14 cells. For isolation of lymphocytes from tissues, anesthetized mice were perfused through the left ventricle with PBS and single-cell suspensions from the lung, spleen, liver, and inguinal lymph nodes were prepared as previously described [27]. Surface marker expression and heterogeneity among *early*M and *late*M P14 cells was determined by 8 color staining of isolated lymphocytes for Thy1.1, CD8, CD27, CD122, KLRG1, CD11b (eBioscience), CD62L, and CD127 (biolegend), or 8 color staining of isolated lymphocytes for Thy1.1, CD8, CD27 (eBioscience), CxCr3, CD127, CCR5, and CD43 (biolegend). Surface marker expression and heterogeneity among endogenous GP_33_ and GP_276_ *early*M and *late*M cells was determined by 8 color staining of isolated splenocytes for GP_33_ or GP_276_ tetramer, Thy1.1, CD8, CD27, CD122, KLRG1 (eBioscience), CD62L and CD127 (biolegend). Cells were acquired on a LSR II instrument (BD Biosciences), and gates were set using fluorescence minus one (FMO) staining. Ex vivo cytokine detection was determined as previously described [74] by mixing splenocytes containing *early*M and *late*M P14 cells and incubating with 200nM GP_33-41_ peptide at 37°C for 5 hours in the presence of Brefeldin A (BFA) for 5 hours or 1 hour. To prevent CD62L cleavage, cells were pre-incubated for ½ hour in the presence of 100μM TAPI-2 (Peptides International) as previously described [75]. To determine functional avidity, splenocytes were incubated as described above in the presence of the indicated concentrations of GP_33-41_ peptide as described previously [76]. To determine the time required to produce IFN-γ, TNF-α, or IL-2, splenocytes containing *early*M and *late*M P14 cells were mixed together and incubated as described above in the presence of 200nM GP_33-41_ peptide for the indicated periods of time. Cells were surface stained for Thy1.1, Thy1.2, CD8, and CD62L (eBioscience), then permeabalized and stained intracellularly for IFN-γ, TNF-α, or IL-2 production. Some samples were also surface stained for expression of CD25, CD69, and CD122. For detection of degranulation, cells were pre-incubated with TAPI-2 for ½ hour then in the presence of 200nM GP_33-41_ peptide plus monensin and anti-CD107a antibodies (BD Biosciences) for 5 hours at 37°C prior to surface staining as previously described [76]. For detection of cycling, cells were pre-incubated with TAPI-2 for ½ hour then in the presence of 200nM GP_33-41_ for 5–24 hours at 37°C. Cells were then surface stained for CD8, Thy1.1, Thy1.2, and CD62L (eBioscience), then permeabilized using a Foxp3 staining kit (ebioscience) and stained intercellularly with Abs against KI67 (BD Pharmingen). Cells were acquired on a FACSCanto instrument (BD Biosciences). For detection of polyfunctional cytokine production, *early*M and *late*M P14 cells from spleens were incubated in the presence of 200nM GP_33-41_ peptide as described above. Cells were surface stained for CD8, Thy1.1, and CD62L (eBioscience) then permeabilized and stained intracellularly for IFN-γ, TNF-α, and IL-2 production. Cells were acquired on a LSR II instrument (BD Biosciences), and gates were set using cells incubated in the absence of GP_33-41_ peptide. To determine expression of Eomes and TCF1, splenocytes were surface stained for CD8, Thy1.1, and CD62L, permeabilized using a Foxp3 staining kit (ebioscience), and stained intercellularly with Abs against Eomes (ebioscience) or TCF1 (Cell signaling).

### Detection of BrdU uptake, responsiveness to IL-15, and homeostatic proliferation in Rag-/- mice

For detection of basal proliferation, mice were i.p. injected with 2mg BrdU and given 0.8 mg/mL BrdU in drinking water for an additional 8 days. P14 cells isolated from peripheral blood were surface stained for CD8 and Thy1.1 (eBioscience) followed by fixation and permeabilization procedures as recommended in the BrdU flow kit (BD Biosciences). Anti-BrdU mAb (eBioscience) was used for intracellular staining to detect BrdU uptake. Cells were acquired on a FACSCalibur instrument (BD Biosciences). For determination of responsiveness to IL-15, splenocytes containing *early*M and *late*M P14 cells were mixed together, washed three times in PBS, and CFSE labeled by incubating 10^7^ splenocytes/mL in room temperature PBS for 15 minutes at 37°C in the presence of 5μM CFSE. CFSE-labeled cells were incubated on ice for 5 minutes with 1mL of fetal calf serum (FCS) and washed three times with RPMI 1640 containing 10% fetal calf serum. CFSE labeled cells were incubated for 3 days at 37°C in the presence or absence of the indicated concentrations of recombinant mouse IL-15 (biolegend). Cells were surface stained for Thy1.1, Thy1.2, CD8, and CD62L (eBioscience) and acquired on a FACSCanto instrument (BD Biosciences). Cells incubated without IL-15 were used to set gates for CFSE dilution. For detection of homeostatic proliferation in Rag-/- mice, P14 cells were isolated from spleens of mice containing Thy disparate *early*M and *late*M P14 cells, and cells were surface stained for Thy1.1, CD8, and CD62L (eBioscience) and sorted using a BD FACSAria II (BD Biosciences). Sorted cells were mixed at a 1:1 ratio and 3x10^4^ of each were transferred retro-orbitally into Rag-/- mice. Sorted cells were >95% pure. Input ratios of *early*M and *late*M P14 cells were confirmed by flow cytometry before adoptive transfer. Rag-/- mice were sacrificed on d15 after transfer, and ratios of *early*M and *late*M P14 cells in spleens were determined by surface staining for Thy1.1, Thy1.2, and CD8. Cells were run on a FACSCalibur instrument (BD Biosciences), and *early*M and *late*M P14 cells were distinguished based on Thy disparity.

### Measure of bacterial and viral clearance

Total splenocytes from mice containing *early*M or *late*M P14 cells were stained with PE-anti-Thy1.1 antibodies and purified with anti-PE magnetic bead sorting with standard AutoMacs protocols. CD62Lhi cells were further surface stained for CD8 and CD62L (ebioscience) and sorted using a BD FACSAria II (BD Biosciences). For determination of protection based on bacterial clearance, 7x10^4^ PE-selected *early*M or *late*M populations or sorted CD62Lhi *early*M or *late*M P14 cells were transferred retro-orbitally into naïve C57BL/6 mice followed 24 hours later by i.v. injection of 1x10^5^ CFU of virulent *Listeria monocytogenes* expressing the GP33 epitope. Three days after infection, spleens were harvested and placed in sterile deionized water containing 0.2% IGEPAL and disrupted using a tissue homogenizer. Samples were plated on tryptic soy broth (TSB)-agar plates containing streptomycin and incubated at 37°C for 24 hours, then CFUs were counted. For determination of protection based on viral clearance, 5x10^4^ PE selected *early*M or *late*M populations or sorted CD62Lhi *early*M or *late*M P14 cells were transferred retro-orbitally into naïve C57BL/6 mice followed 24 hours later by i.v. infection of 2x10^6^ PFU of LCMV clone-13. 8 days after infection spleens were obtained and homogenized, and viral titers were quantified with standard plaque assaying on VERO cells as previously described [10]. Sorted cells were >95% pure. Control naïve mice did not receive adoptive transfer of P14 cells.

### Microarray data acquisition and analysis

CD62Lhi *early*M and *late*M P14 cells were isolated from spleens and cells were surface stained for Thy1.1, CD8, and CD62L (eBioscience) and sorted using a BD FACSAria II (BD Biosciences). Samples from three individual mice were obtained for each group, and sorted cells were >95% pure. RNA was extracted using the RNEasy Kit (QIAGEN), and 1-5ng of mRNA was used for microarray analysis. RNA quality was assessed using the Agilent Model 2100 Bioanalyzer. mRNA for the microarray was processed using the NuGEN WT-Ovation Pico RNA Amplification System along with the NuGEN WT-Ovation Exon Module. Samples were hybridized and loaded onto Affymetrix GeneChip Mouse 1.0 ST arrays. Arrays were scanned with the Affymetrix Model 7G upgraded scanner, and data were collected using the GeneChip Operating Software. Data from the Affymetrix Mouse Exon 1.0 ST arrays were first quantile normalized and median polished using Robust Multichip Average background correction with log2 adjusted values. Probe sets for exons were then summarized for a specific gene using the median value. After obtaining log2 expression values for genes, significance testing was performed using analysis of variance (ANOVA). Functional assignment of genes was performed using the “Functional Annotation Tools” in DAVID bioinformatics resources (https://david.ncifcrf.gov) following recommended protocols [38]. Enrichment of genes in known pathways was analyzed using the KEGG pathway tool, and GSEA was performed as described [51,77]. The microarray data were deposited in the NCBI Gene Expression Omnibus with the accession number GSE63307.

### Quantitative RT-PCR

Total splenocytes from mice containing *early*M or *late*M P14 cells were stained with PE-anti-Thy1.1 antibodies and purified with anti-PE magnetic bead sorting with standard AutoMacs protocols. CD62Lhi cells were further surface stained for CD8 and CD62L (ebioscience) and sorted using a BD FACSAria II (BD Biosciences). Sorted CD62Lhi *early*M and *late*M cells were then incubated with or without 200nM GP_33-41_ peptide at 37°C for 5 hours. Total RNA was reverse-transcribed using a QuantiTech Reverse Transcription Kit (Qiagen). The resulting cDNA was analyzed for expression of different genes by quantitative PCR using SYBR Advantage qPCR pre-mix (Clontech) on an ABI 7300 Real Time PCR System (Applied Biosystems). Relative gene expression levels in each sample were normalized to that of a housekeeping gene, hypoxanthine phosphoribosyltranserase 1 (*Hprt1*) [62].

The primers used in quantitative RT-PCR were as follows:

*Ifng*: 5’-GCGTCATTGAATCACACCTG and 3’-TGAGCTCATTGAATGCTTGG; *Tnfa*: 5’-TAGCCCACGTCGTAGCAAAC and 3’-GCAGCCTTGTCCCTTGAAGA; *Il2*: 5’-AACCTGAAACTCCCCAGGAT and 3’-CGCAGAGGTCCAAGTTCATC; *Xcl1*: 5’-ATGGGTTGTGGAAGGTGTGG and 3’-TGATCGCTGCTTTCACCCAT; *Ccl3*: 5’-CATATGGAGCTGACACCCCG and 3’-GTCAGGAAAATGACACCTGGC; *Ccl5*: 5’-GACAGCACATGCATCTCCCA and 3’-GTGTCCGAGCCATATGGTGA; *Fasl*: 5’-GCAGAAGGAACTGGCAGAAC and 3’-TTAAATGGGCCACACTCCTC;

*Klrg1*: 5’-TCCTCTGGACGAGGAATGGT and 3’-ACAGCTTCACTCCCTGGTTG; *Il2ra*: 5’-GGTGCATAGACTGTGTTGGC and 3’-GCAAGAGAGGTTTCCGAAGAC; *Cd69*: 5’-ACATCTGGAGAGAGGGCAGA and 3’-AAGGACGTGATGAGGACCAC; *Ccr5*: 5’-CCCCTACAAGAGACTCTGGCTC and 3’-TTTTGGCAGGGTGCTGACAT; *Il12rb2*: 5’-GTGTCTGCAGCCAACTCAAA and 3’-AGGCTGCCAGGTCACTAGAA; *Il18rap*: 5’-GCAGGCTTACTCACCATTTCA and 3’-GCTTGTGCATCTTTATCCACGG; *Cx3cr1*: 5’-AAGTTCCCTTCCCATCTGCT and 3’-CGAGGACCACCAACAGATTT; *Tbx21*: 5’-TCAACCAGCACCAGACAGAG and 3’-CCACATCCACAAACATCCTG;

*Eomes*: 5’-GGAAGTGACAGAGGACGGTG and 3’-AGCCGTGTACATGGAATCGT; *Tcf7*: 5’-CAATCTGCTCATGCCCTACC and 3’-CTTGCTTCTGGCTGATGTCC; *Prdm1*: 5’-CCTGCCAACCAGGAACTTCT and 3’-GTTGCTTTCCGTTTGTGTGAGA; *Foxm1*: 5’-CGAGCACTTGGAATCACAGC and 3’-GGATGGGCACCAGGTATGAG; *Id2*: 5’-CATCAGCATCCTGTCCTTGC and 3’-GTGTTCTCCTGGTGAAATGG;

*Id3*: 5’-TGATCTCCAAGGACAAGAGGA and 3’-TGAAGAGGGCTGGGTTAAGA; *Bcl2*: 5’-GGAGGCTGGGATGCCTTTGT and 3’-TGCACCCAGAGTGATGCAG; *Bcl6*: 5’-CCTGAGGGAAGGCAATATCA and 3’-CGGCTGTTCAGGAACTCTTC; *Foxo3*: 5’-CTCATGGATGCTGACGGGTT and 3’-CGTCAGTTTGAGGGTCTGCT; *Stat3*: 5’-TGGTGTCCAGTTTACCACGA and 3’-TGTTCGTGCCCAGAATGTTA; *Stat4*: 5’-TTTTGACGCTGCAAGAAATG and 3’-TCCAGTCCTGCAGCTCTTCT; *Myc*: 5’-GTACCTCGTCCGATTCCACG and 3’-GCCTCTTCTCCACAGACACC;

*Ccnd2*: 5’-TCAGTGTGGGTGATCTTGGC and 3’-CAGACCTTCATCGCTCTGTG; *Ccnd3*: 5’-GGACACTCGCTTTGTTTGGG and 3’-AGCATTTCAGGGCGAGCTTA; *Ccne1*: 5’-GTGGAGCTTATAGACTTCGCAC and 3’-ACTTACCTGAGAGATGAGCACT; *Ccne2*: 5’-AGAGTCGATGGCTAGAATGC and 3’-TGTCCAGTAACAGTCATCTCCT; *Ccna2*: 5’-GGTGAAGGCAGGCTGTTTAC and 3’-AGAAGCTCAAGACTCGACGG; *Ccnb1*: 5’-CCTGAGCCTGAACCTGAACT and 3’-ACGTCACTCACTGCAAGGAT; *Ccnb2*: 5’-GCAGAGCAGAGCATCAGAGA and 3’-CAGCCTCTGTGAAACCAGTG; *Cdk1*: 5’-TCAAGTCTCTGTGAAGAACTCG and 3’-TCCATGGACCTCAAGAAGTACC; *Cdk2*: 5’-CAATGCAGAGGGGTCCATCA and 3’-ACACACTAGGTGCATTTCAGC; *Cdk4*: 5’- CAGGTAGGAGTGCTGCAGG and 3’-AGTCAGTGGTGCCAGAGATG; *Cdk5*: 5’-GGATCTTCCGACTGCTAGGG and 3’-GCTGCACAGGGTTACACTTC;

*Cdk6*: *5’-*GCATCGTGATCTGAAACCGC and 3’-GTGACGACCACCGAGGTAAG.

### Metabolism assays

Both *early*M and *late*M populations and CD62Lhi subsets were analyzed for metabolic function. Populations of *early*M and *late*M cells were isolated from total splenocytes from mice containing *early*M or *late*M P14 cells by staining with PE-anti-Thy1.1 antibodies and purifying with anti-PE magnetic bead sorting using standard AutoMacs protocols. Additional cells were sorted for CD62Lhi subsets after AutoMacs purification by surface staining for CD8 and CD62L (ebioscience) and sorting using a BD FACSAria II (BD Biosciences). 2x10^5^ purified earlyM or *late*M populations or CD62Lhi cells were plated in XF media, and oxygen consumption rates (OCR) and extracellular acidification rates (ECAR) were measured under basal conditions and in response to 1 μM oligomycin, 1.5μM fluorocarbonyl cyanide phenylhydrazone (FCCP), and 0.5 μM rotenone + 1μM antimycin with the XF-96 Extracellular Flux Analyzer (Seahorse Bioscience) [46].

### Accession numbers (Entrez Gene)

Sell (CD62L) ID:20343, CD27 ID:21940, CCR7 ID:12775, Thy1 ID:21838, IL7R (CD127) ID:16197, IL2Rb (CD122) ID:16185, KLRG1 ID:50928, IFNg ID:15978, TNFa ID:21926, Lamp1 (CD107a) ID:16783, IL2 ID:16183, IL15 ID:16168, Itgam (CD11b) ID:16409, CxCr3 ID:12766, CCR5 ID:12774, IL15ra ID:16169, PRF1 (perforin) ID:18646, GZMB (granzymeB) ID:3002, Il2rg ID:16186, Itgal (CD11b) ID:16408, CD44 ID:12505, Anapc5 ID:59008, Bub1 ID:12235, Ccnb2 ID:12442, Ccne2 ID:12448, Ccnh ID:66671, Cdc7 ID:12545, Cdk1 ID:12534, Cdk4 ID:12567, Cdkn2c ID:12580, Mad2l1 ID:56150, Orc6l ID:56452, Rb1 ID:19645, Skp1a ID:21402, Ttk ID:22137, Abl1 ID:11350, Ccnd3-ps ID:626000, Cdc25b ID:12531, Gadd45g ID: 23882, Zbtb17 ID:22642, Atp5e ID:67126, Atp5f1 ID:11950, Atp5g2 ID:67942, Atp5l ID:27425, Atp5o ID:28080, Atp6v0c ID:11984, Atp6v1e1 ID:11973, Atp6v1f ID:66144, Atpgv1g1 ID:66290, Cox4i1 ID:12857, Cox6a1 ID:12861, Cox7b ID:66142, Ndufa2 ID:17991, Ndufa11 ID:69875, Ndufb6 ID:230075, Ndufb8 ID:67264, Ndufc2 ID:68197, Ndufv1 ID:17995, Ndufv2 ID:72900, Sdhb ID:67680, Sdhc ID:66052, Uqcr1 ID:7384, Uqcrq ID:22272, Atp4b ID:11945, Atp6v0e2 ID:76252, IL2ra ID:16184, CD69 ID:12515, IL12rb2 ID:16162, IL18rap ID:16174, Cx3Cr1 ID:13051, GZMA (granzymeA) ID:14938, GZMK (granzymeK) ID:14945, Xcl1 ID:16963, Ccl3 ID:20302, Ccl5 ID:20304, Fasl ID:14103, Tbx21 (Tbet) ID:57765, Eomes ID:13813, Tcf7 (Tcf1) ID:21414, Prdm1 (blimp1) ID:12142, Foxm1 ID:14235, Id2 ID:15902, Id3 ID:15903, Bcl2 ID:12043, Bcl6 ID:12053, Foxo3 ID:56484, Stat3 ID:20848, Stat4 ID:20849, Myc ID:17869, Ccnd2 ID:12444, Ccnd3 ID:12445, Ccne1 ID:12447, Ccna2 ID: 12428, Ccnb1 ID: 268697, Cdk2 ID:12566, Cdk5 ID:12568, Cdk6 ID:12571

## Supporting Information

S1 Fig Phenotype of endogenous memory CD8 T cell populations and CD62Lhi subsets changes, and phenotypic heterogeneity decreases with time after infection. (A-D) splenocytes from mice infected with LCMV 30–45 days (*early*M) or 8+ months (*late*M) previously were co-stained for GP_33_ or GP_276_ tetramer, CD8, Thy1.1, CD62L, CD27, CD122, CD127, and KLRG1. (A) Representative histograms showing CD127, CD62L, CD27, CD122, and KLRG1 expression on gated *early*M (open histograms) and *late*M (grey histograms) endogenous GP_33_ or GP_276_ tetramer positive cells isolated from spleens. (B) Representative histograms of CD127, CD27, CD122, and KLRG1 expression on gated CD62Lhi endogenous GP_33_ or GP_276_ tetramer positive *early*M (open histogram) and *late*M (grey histogram) cells in the spleen. (C) Percentages of subpopulations out of total CD62Lhi endogenous GP_33_ or GP_276_ tetramer positive *early*M and *late*M cells. Surface marker expression patterns for the 16 possible subpopulations are indicated in the figure legend. (D) Number of subpopulations (out of 16 possible) comprising greater than 1% of the total CD62Lhi endogenous GP_33_ or GP_276_ population for *early*M and *late*M cells in the spleen. Representative data from one of two individual experiments with 3 mice per group per experiment. Error bars represent the standard error of the mean.
(TIFF)

S2 Fig With time, memory CD8 T cells convert to CD62Lhi and become enriched for T_DIM_S. Analysis was performed at the indicated days post LCMV infection or on CD62Lhi *early*M (30–45 days p.i.) and *late*M (8+ months p.i.) P14 cells. (A) Representative histograms showing CD62L expression on gated P14 cells isolated from spleens at the indicated days p.i. (B) Representative histograms showing IFN-γ production by gated CD62L- or CD62Lhi *early*M (open histograms) or *late*M (shaded histograms) P14 cells isolated from spleens as determined by ICS following 5hr incubation with or without GP_33-41_ peptide. Numbers inside plots indicate the percentage of *early*M (top) or *late*M (bottom) cells not producing IFN-γ. Representative data from one of three individual experiments with 3 mice per group per experiment.
(TIFF)

S3 Fig Subpopulations of CD62L+ *early*M and *late*M P14 cells. *early*M (30–45 days p.i.) and *late*M (8+ months p.i.) P14 cells from the indicated organs. (A) Cells were co-stained for CD62L, CD27, CD122, CD127, KLRG1, and CD11b. Percentages of subpopulations out of total CD62Lhi *early*M or *late*M P14 cells. (B) Cells were co-stained for CD62L, CD27, CxCr3, CD127, CCR5, and CD43. Percentages of subpopulations out of total CD62Lhi *early*M or *late*M P14 cells. Surface marker expression patterns for the 32 possible subpopulations are indicated in the figure legend. Representative data from one of three individual experiments with 3 mice per group per experiment. Error bars represent the standard error of the mean.
(TIFF)

S4 Fig CD62Lhi *early*M and *late*M cells exhibit a similarly poised state for effector responses, but a heightened state for proliferation with time after infection based upon flow cytometric analysis following Ag re-encounter. (A-D) *early*M and *late*M P14 cells were mixed and incubated for 5 (A-C) or 5–24 (D) hours in the presence (+) or absence (-) of GP_33-41_ peptide. (A) Percentage of *early*M or *late*M P14 cells producing the indicated cytokines. (B) Percentage of *early*M or *late*M P14 cells expressing the indicated surface markers. (C) Percentage of *early*M or *late*M P14 cells expressing (top), and per cell expression based on gMFI (bottom), of the indicated transcription factors. (D) Percentage of *early*M or *late*M P14 cells staining positive for KI67 following incubation with GP_33-41_ peptide for the indicated lengths of time. Data from one experiment with 3 mice per group per experiment. Error bars represent the standard error of the mean.
(TIFF)

S5 Fig CD62Lhi *early*M and *late*M cells exhibit a similarly poised state for recall responses based upon mRNA expression following Ag re-encounter. (A-D) mRNA was extracted from sorted CD62Lhi *early*M and *late*M P14 cells that were incubated for 5 hours in the presence (+) or absence (-) of GP_33-41_ peptide. (A) mRNA expression of the indicated effector molecules. (B) mRNA expression of the indicated surface/activation markers. (C) mRNA expression of the indicated transcription factors. (D) mRNA expression of the indicated cell cycle associated genes. Expression is relative to HPRT1. Data from one experiment with 3 mice per group per experiment. Error bars represent the standard error of the mean.
(TIFF)

S6 Fig Cell cycle pathways are differentially regulated in CD62Lhi memory CD8 T cells with time. mRNA was isolated from sorted CD62Lhi *early*M (30–45 days p.i.) and *late*M (8+ months p.i.) P14 cells and used for microarray hybridization. (A) Biological pathway analysis of genes with significant mRNA changes of fold >1.25 in CD62Lhi *early*M and *late*M P14 cells was generated using the KEGG pathway tool in DAVID bioinformatics resources. Shown is relative gene expression for genes involved in the KEGG cell cycle pathway that are differentially expressed between CD62Lhi *early*M and *late*M P14 cells. Positive fold changes represent genes with increased expression in CD62Lhi *late*M compared to *early*M P14 cells while negative fold changes represent genes with decreased expression in CD62Lhi *late*M compared to *early*M P14 cells. (B) Gene set enrichment analysis was performed comparing expression of genes in CD62Lhi *early*M and *late*M P14 cells to existing gene sets. *late*M P14 cells showed enrichment for several gene sets involved in cell cycling.
(TIFF)

S7 Fig Ability to undergo Ag-driven proliferation increases in endogenous memory CD8 T cells with time after infection. (A) Experimental design. 2.5x10^3^ endogenous GP_276_ tetramer positive cells among sorted CD62Lhi CD8 T cells from the spleens of mice infected 1 month (*early*M) or >8 months (*late*M) previously with LCMV were transferred into naïve C57B6/SJL mice. Recipient mice were infected 24 hours later with LCMV. (B) Representative dot plots from the spleens of recipient mice 7 days post LCMV infection showing gating of transferred cells (CD45.2) and 2° effector GP_276_+ cells generated from transferred CD62Lhi 1° *early*M or *late*M GP_276_+ cells. (C) (left) Percentage of 2° effector cells out of total lymphocytes generated from transferred CD62Lhi *early*M or *late*M endogenous GP_276_ tetramer positive cells in PBL of recipient mice 7 days post LCMV infection. (right) Total numbers of 2° effector cells generated from transferred CD62Lhi *early*M or *late*M endogenous GP_276_ tetramer positive cells recovered from the spleens of recipient mice 7 days post LCMV infection. Data from one to two individual experiments with 3–5 mice per group per experiment. Error bars represent the standard error of the mean.
(TIFF)

S8 Fig Eomes and TCF1 expression differs in populations, but not CD62Lhi subsets of *early*M and *late*M cells. (A) Representative histograms of Eomes (top) and TCF1 (bottom) expression in gated whole populations (left) or CD62Lhi (right) *early*M (open histogram) and *late*M (grey histogram) P14 cells.
(TIFF)
