# Supplementary figures and images for: Phenotypic and Functional Alterations in Circulating Memory CD8 T Cells with Time after Primary Infection
Source: PLoS Pathog. 2015 Oct 20;11(10):e1005219. doi: 10.1371/journal.ppat.1005219 (PMC4618693; doi:10.1371/journal.ppat.1005219)

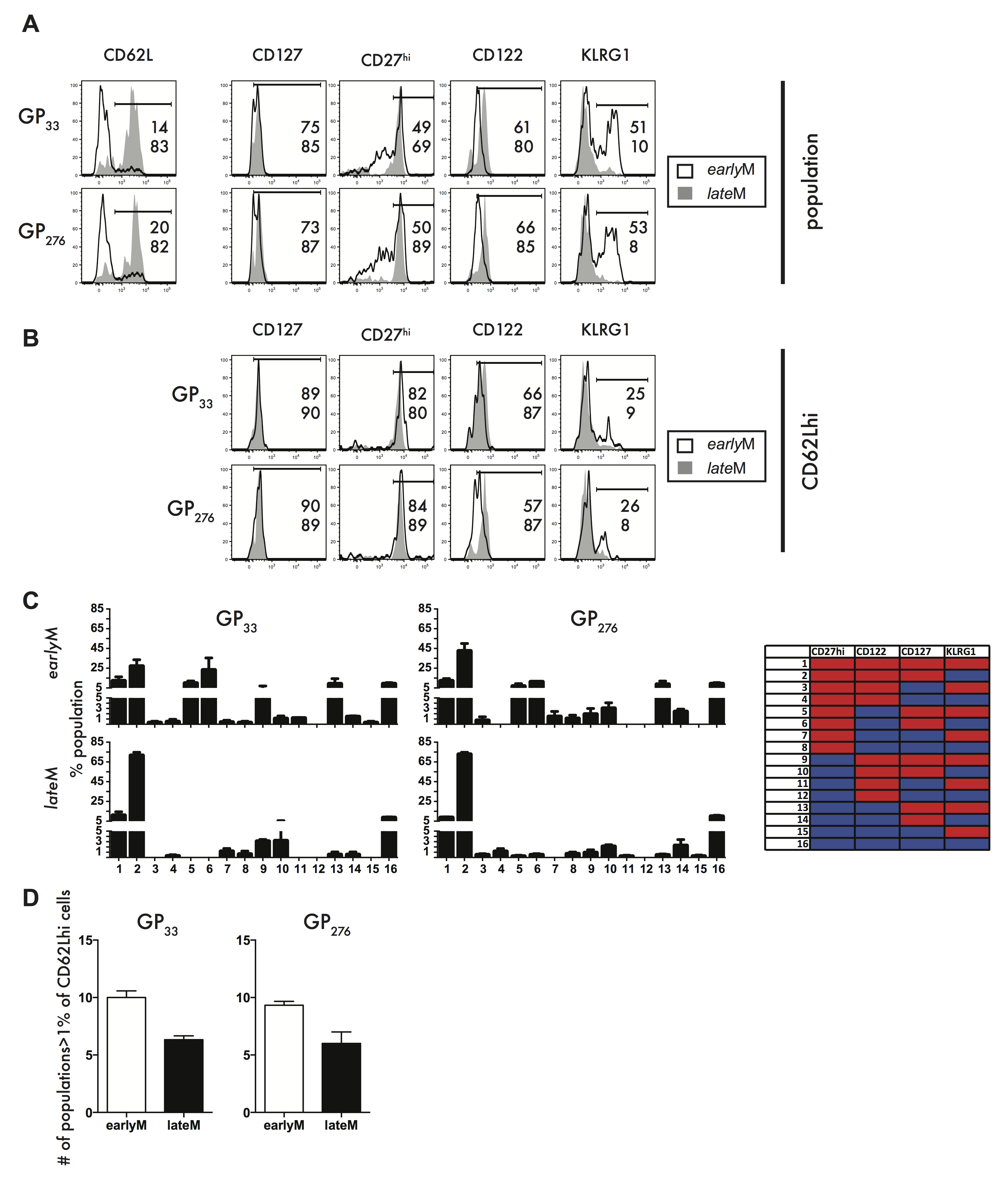

Supplement: S1 Fig — (A-D) splenocytes from mice infected with LCMV 30–45 days (earlyM) or 8+ months (lateM) previously were co-stained for GP33 or GP276 tetramer, CD8, Thy1.1, CD62L, CD27, CD122, CD127, and KLRG1. (A) Representative histograms showing CD127, CD62L, CD27, CD122, and KLRG1 expression on gated earlyM (open histograms) and lateM (grey histograms) endogenous GP33 or GP276 tetramer positive cells isolated from spleens. (B) Representative histograms of CD127, CD27, CD122, and KLRG1 expression on gated CD62Lhi endogenous GP33 or GP276 tetramer positive earlyM (open histogram) and lateM (grey histogram) cells in the spleen. (C) Percentages of subpopulations out of total CD62Lhi endogenous GP33 or GP276 tetramer positive earlyM and lateM cells. Surface marker expression patterns for the 16 possible subpopulations are indicated in the figure legend. (D) Number of subpopulations (out of 16 possible) comprising greater than 1% of the total CD62Lhi endogenous GP33 or GP276 population for earlyM and lateM cells in the spleen. Representative data from one of two individual experiments with 3 mice per group per experiment. Error bars represent the standard error of the mean. (TIFF) [file ppat.1005219.s001.tiff]

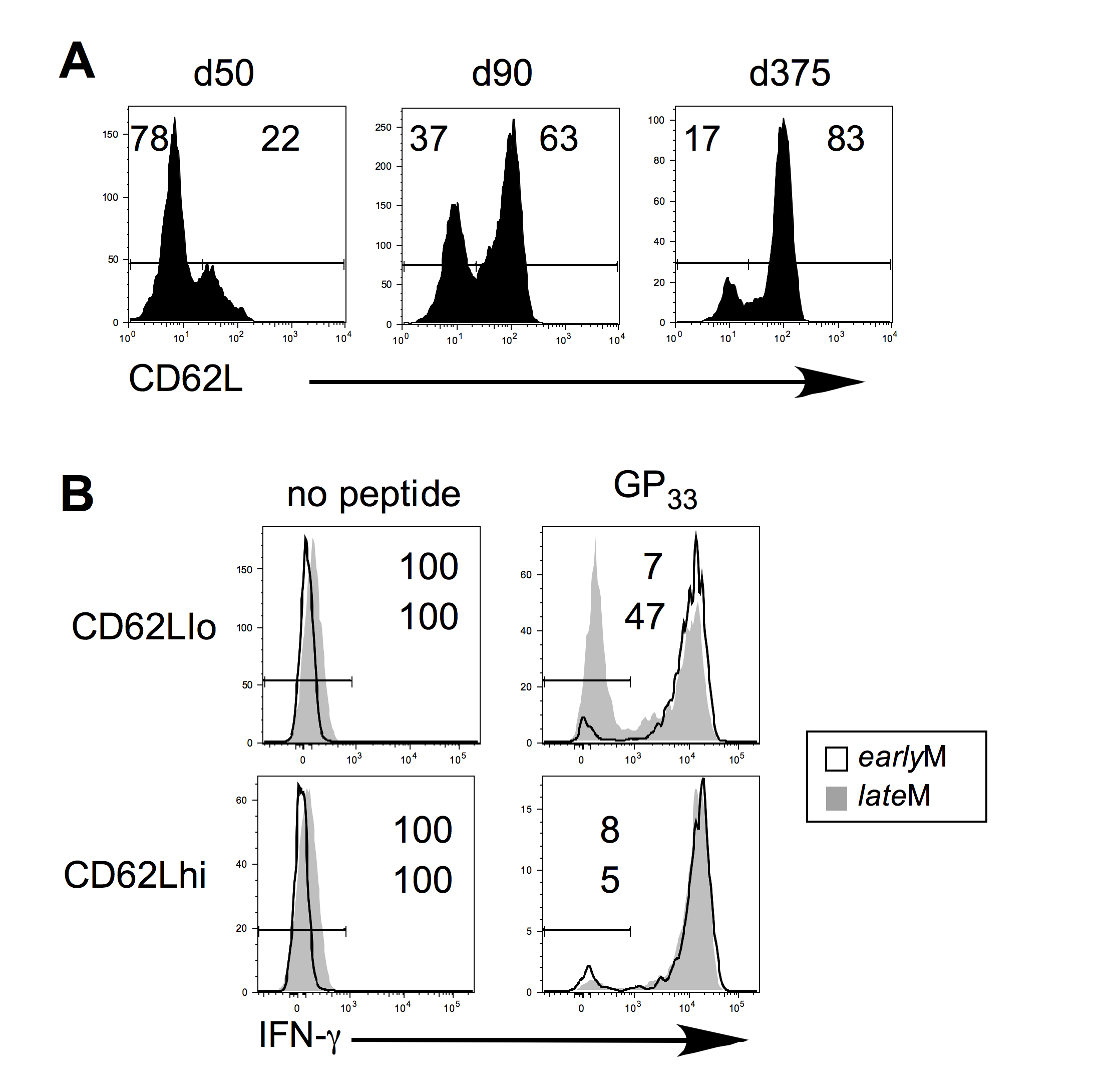

Supplement: S2 Fig — Analysis was performed at the indicated days post LCMV infection or on CD62Lhi earlyM (30–45 days p.i.) and lateM (8+ months p.i.) P14 cells. (A) Representative histograms showing CD62L expression on gated P14 cells isolated from spleens at the indicated days p.i. (B) Representative histograms showing IFN-γ production by gated CD62L- or CD62Lhi earlyM (open histograms) or lateM (shaded histograms) P14 cells isolated from spleens as determined by ICS following 5hr incubation with or without GP33-41 peptide. Numbers inside plots indicate the percentage of earlyM (top) or lateM (bottom) cells not producing IFN-γ. Representative data from one of three individual experiments with 3 mice per group per experiment. (TIFF) [file ppat.1005219.s002.tiff]

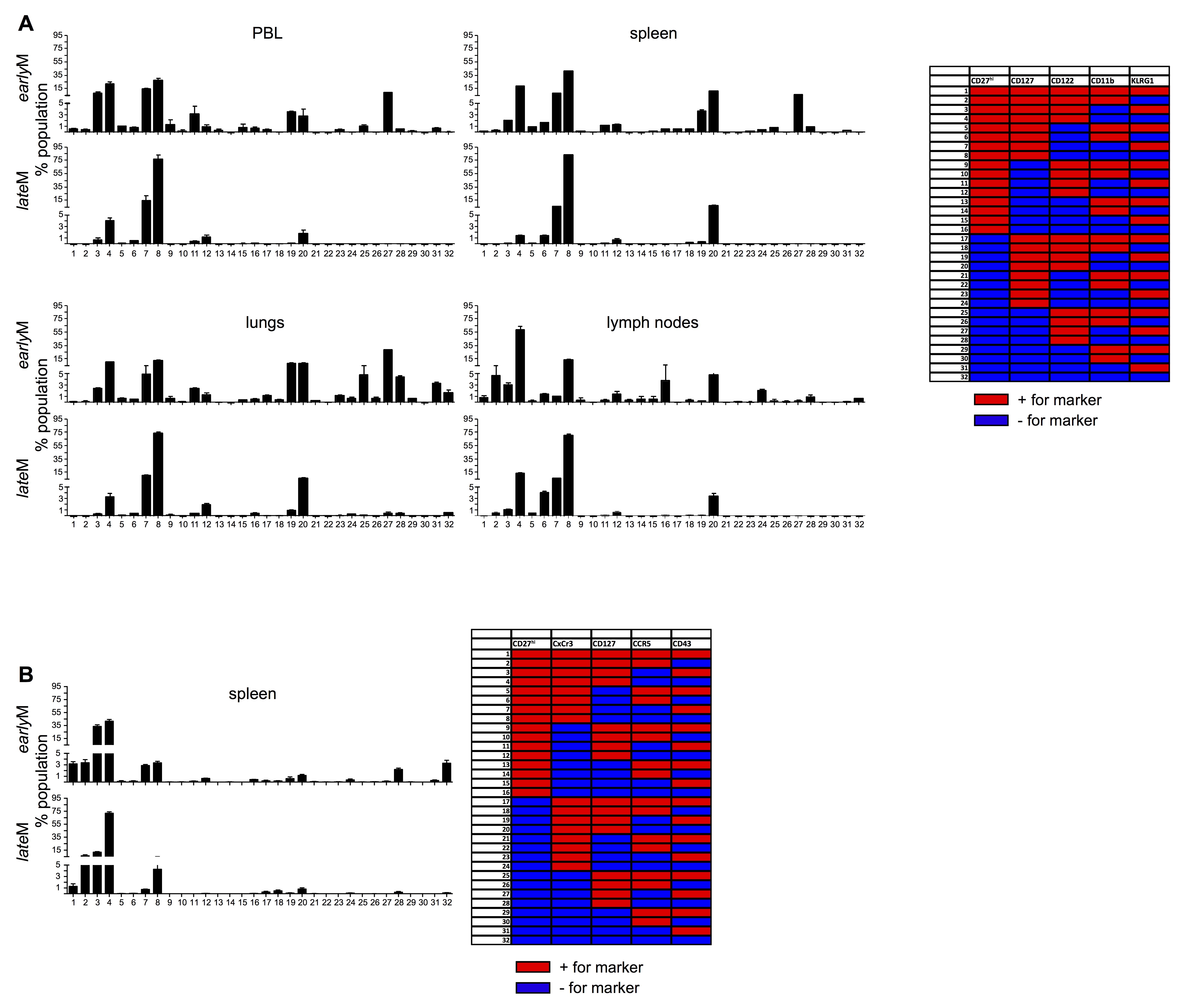

Supplement: S3 Fig — earlyM (30–45 days p.i.) and lateM (8+ months p.i.) P14 cells from the indicated organs. (A) Cells were co-stained for CD62L, CD27, CD122, CD127, KLRG1, and CD11b. Percentages of subpopulations out of total CD62Lhi earlyM or lateM P14 cells. (B) Cells were co-stained for CD62L, CD27, CxCr3, CD127, CCR5, and CD43. Percentages of subpopulations out of total CD62Lhi earlyM or lateM P14 cells. Surface marker expression patterns for the 32 possible subpopulations are indicated in the figure legend. Representative data from one of three individual experiments with 3 mice per group per experiment. Error bars represent the standard error of the mean. (TIFF) [file ppat.1005219.s003.tiff]

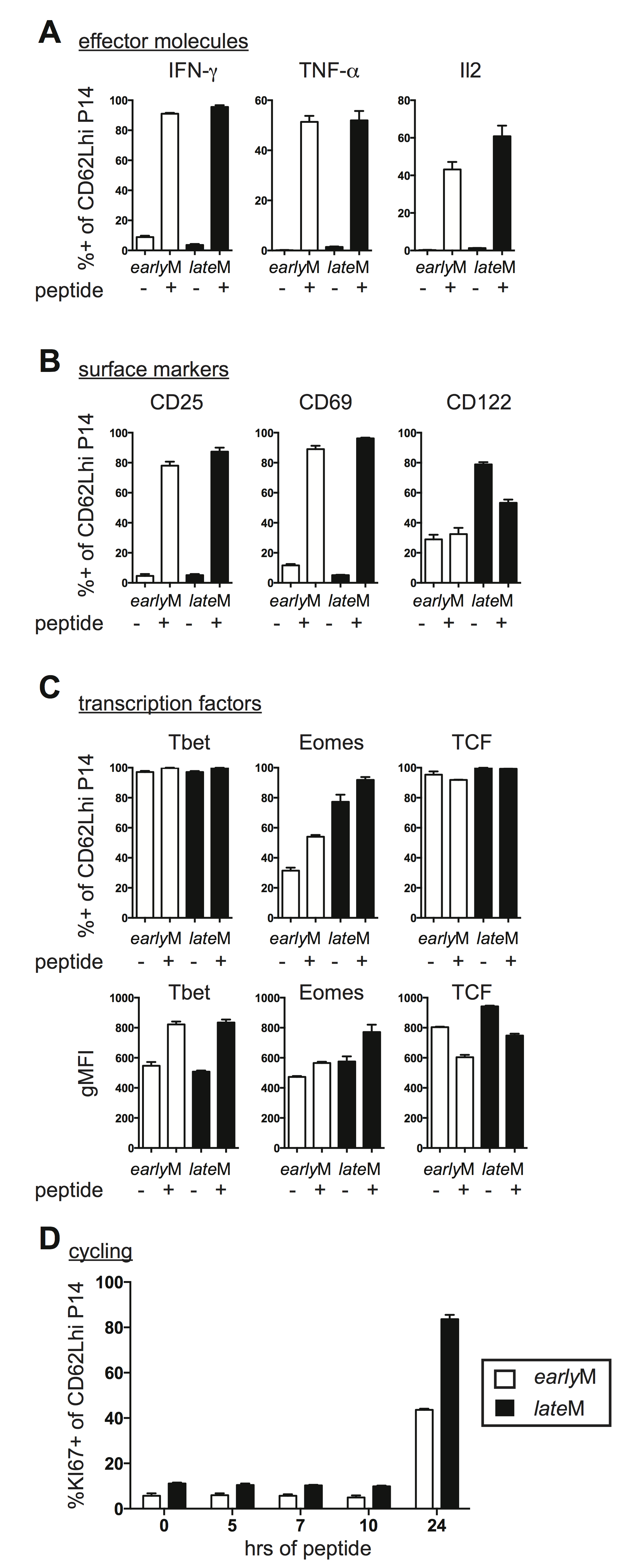

Supplement: S4 Fig — (A-D) earlyM and lateM P14 cells were mixed and incubated for 5 (A-C) or 5–24 (D) hours in the presence (+) or absence (-) of GP33-41 peptide. (A) Percentage of earlyM or lateM P14 cells producing the indicated cytokines. (B) Percentage of earlyM or lateM P14 cells expressing the indicated surface markers. (C) Percentage of earlyM or lateM P14 cells expressing (top), and per cell expression based on gMFI (bottom), of the indicated transcription factors. (D) Percentage of earlyM or lateM P14 cells staining positive for KI67 following incubation with GP33-41 peptide for the indicated lengths of time. Data from one experiment with 3 mice per group per experiment. Error bars represent the standard error of the mean. (TIFF) [file ppat.1005219.s004.tiff]

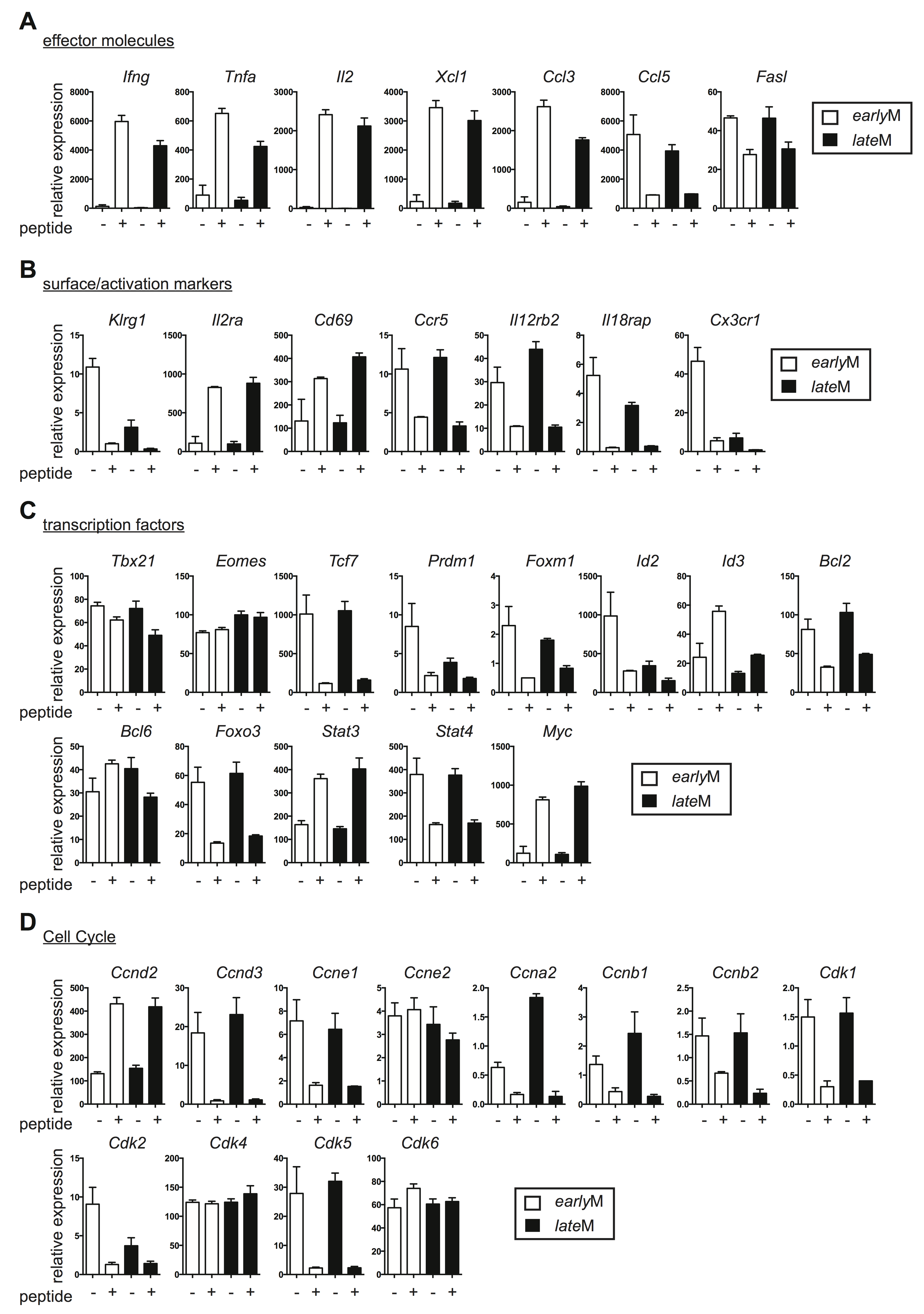

Supplement: S5 Fig — (A-D) mRNA was extracted from sorted CD62Lhi earlyM and lateM P14 cells that were incubated for 5 hours in the presence (+) or absence (-) of GP33-41 peptide. (A) mRNA expression of the indicated effector molecules. (B) mRNA expression of the indicated surface/activation markers. (C) mRNA expression of the indicated transcription factors. (D) mRNA expression of the indicated cell cycle associated genes. Expression is relative to HPRT1. Data from one experiment with 3 mice per group per experiment. Error bars represent the standard error of the mean. (TIFF) [file ppat.1005219.s005.tiff]

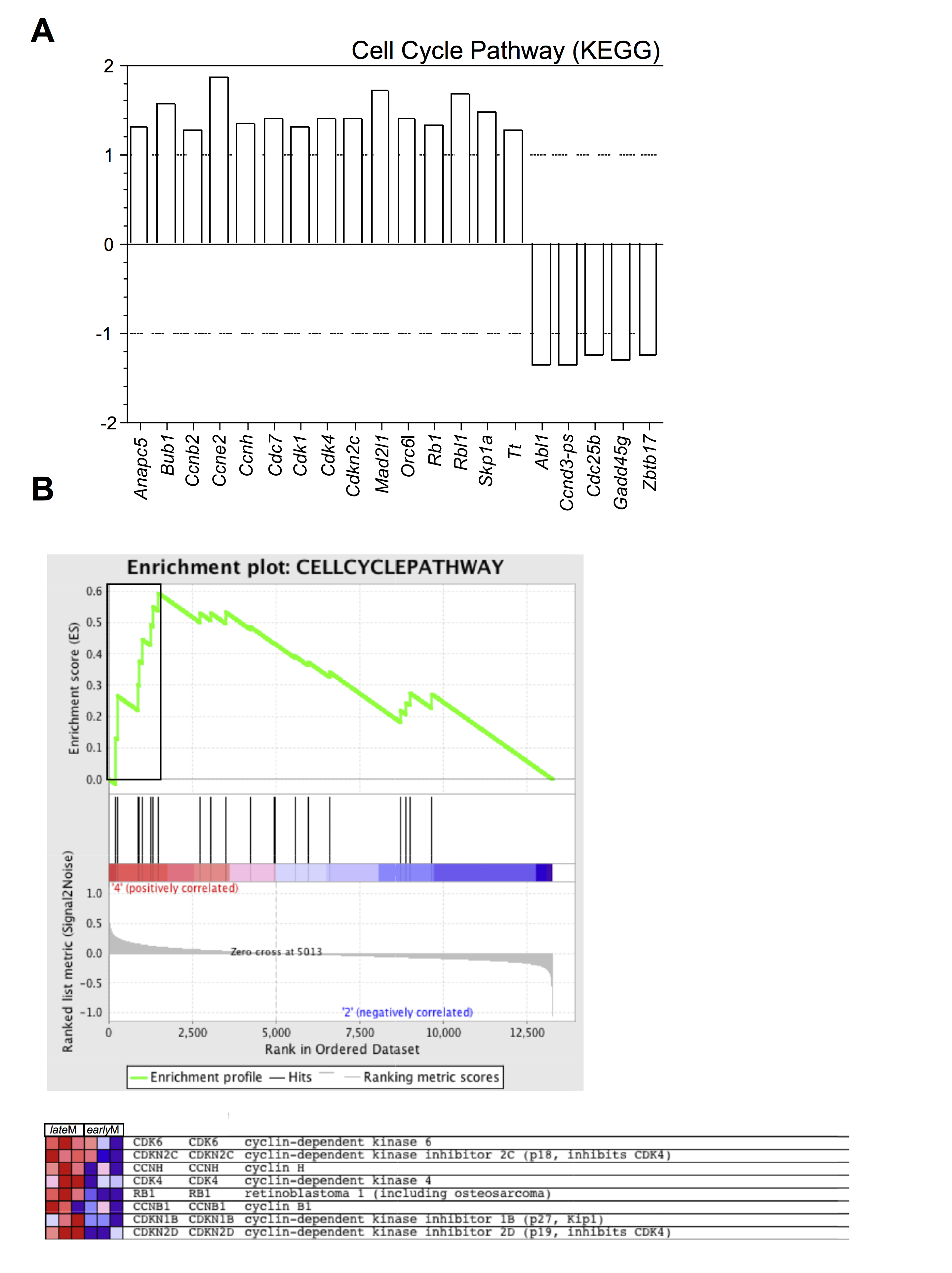

Supplement: S6 Fig — mRNA was isolated from sorted CD62Lhi earlyM (30–45 days p.i.) and lateM (8+ months p.i.) P14 cells and used for microarray hybridization. (A) Biological pathway analysis of genes with significant mRNA changes of fold >1.25 in CD62Lhi earlyM and lateM P14 cells was generated using the KEGG pathway tool in DAVID bioinformatics resources. Shown is relative gene expression for genes involved in the KEGG cell cycle pathway that are differentially expressed between CD62Lhi earlyM and lateM P14 cells. Positive fold changes represent genes with increased expression in CD62Lhi lateM compared to earlyM P14 cells while negative fold changes represent genes with decreased expression in CD62Lhi lateM compared to earlyM P14 cells. (B) Gene set enrichment analysis was performed comparing expression of genes in CD62Lhi earlyM and lateM P14 cells to existing gene sets. lateM P14 cells showed enrichment for several gene sets involved in cell cycling. (TIFF) [file ppat.1005219.s006.tiff]

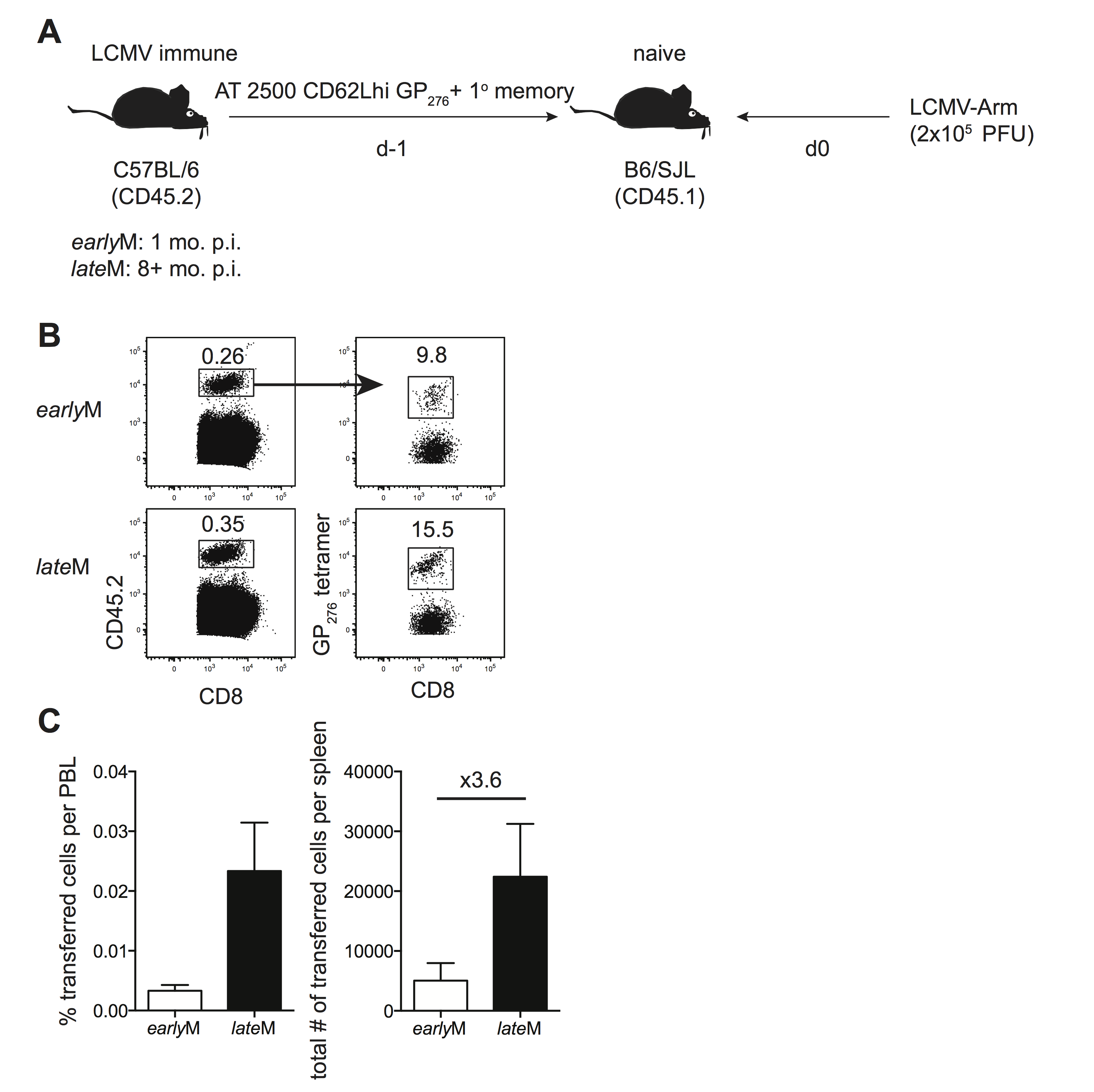

Supplement: S7 Fig — (A) Experimental design. 2.5x10^3^ endogenous GP276 tetramer positive cells among sorted CD62Lhi CD8 T cells from the spleens of mice infected 1 month (earlyM) or >8 months (lateM) previously with LCMV were transferred into naïve C57B6/SJL mice. Recipient mice were infected 24 hours later with LCMV. (B) Representative dot plots from the spleens of recipient mice 7 days post LCMV infection showing gating of transferred cells (CD45.2) and 2° effector GP276+ cells generated from transferred CD62Lhi 1° earlyM or lateM GP276+ cells. (C) (left) Percentage of 2° effector cells out of total lymphocytes generated from transferred CD62Lhi earlyM or lateM endogenous GP276 tetramer positive cells in PBL of recipient mice 7 days post LCMV infection. (right) Total numbers of 2° effector cells generated from transferred CD62Lhi earlyM or lateM endogenous GP276 tetramer positive cells recovered from the spleens of recipient mice 7 days post LCMV infection. Data from one to two individual experiments with 3–5 mice per group per experiment. Error bars represent the standard error of the mean. (TIFF) [file ppat.1005219.s007.tiff]

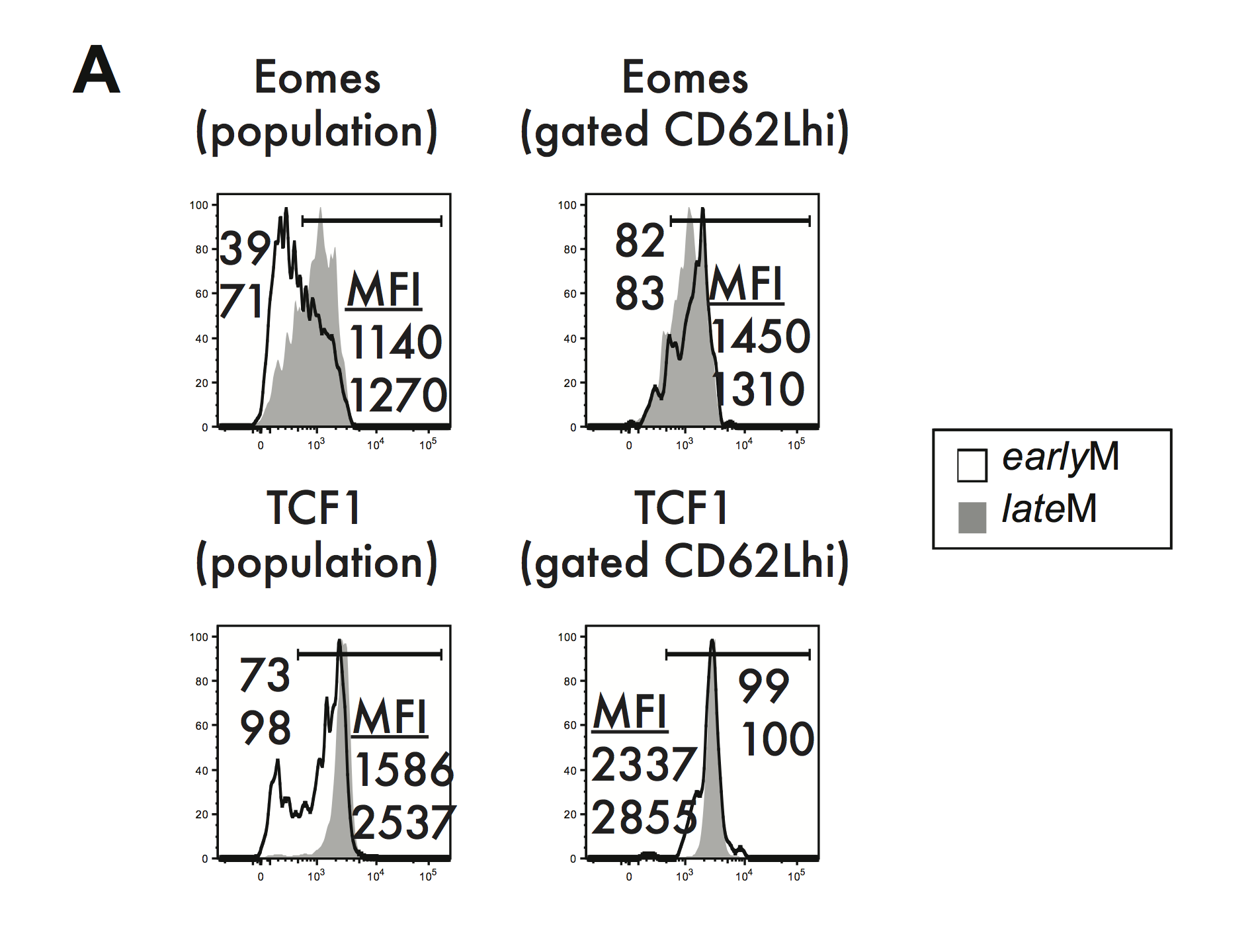

Supplement: S8 Fig — (A) Representative histograms of Eomes (top) and TCF1 (bottom) expression in gated whole populations (left) or CD62Lhi (right) earlyM (open histogram) and lateM (grey histogram) P14 cells. (TIFF) [file ppat.1005219.s008.tiff]
